# The Instruments and Capabilities of the *Miniature X-Ray Solar Spectrometer* (MinXSS) CubeSats

**DOI:** 10.1007/s11207-018-1243-3

**Published:** 2018-01-23

**Authors:** Christopher S. Moore, Amir Caspi, Thomas N. Woods, Phillip C. Chamberlin, Brian R. Dennis, Andrew R. Jones, James P. Mason, Richard A. Schwartz, Anne K. Tolbert

**Affiliations:** 1 Department for Astrophysical and Planetary Science, University of Colorado, Boulder, CO USA; 2 Laboratory for Atmospheric and Space Physics, University of Colorado, Boulder, CO USA; 3 Present Address: Harvard-Smithsonian Center for Astrophysics, Cambridge, MA USA; 4 Southwest Research Institute, Boulder, CO USA; 5 NASA Goddard Space Flight Center, Code 671.0, Greenbelt, MD USA; 6 American University, Washington, DC USA

**Keywords:** CubeSat, Sun, Stars, Corona, X-rays, Spectrometer, Photometer, Quiet Sun, Active Region, Flares, Dynamics, Photon Flux, Magnetic fields, Emission Measure, Temperature, Earth, Thermosphere

## Abstract

The *Miniature X-ray Solar Spectrometer* (MinXSS) CubeSat is the first solar science oriented CubeSat mission flown for the NASA Science Mission Directorate, with the main objective of measuring the solar soft X-ray (SXR) flux and a science goal of determining its influence on Earth’s ionosphere and thermosphere. These observations can also be used to investigate solar quiescent, active region, and flare properties. The MinXSS X-ray instruments consist of a spectrometer, called X123, with a nominal 0.15 keV full-width at half-maximum (FWHM) resolution at 5.9 keV and a broadband X-ray photometer, called XP. Both instruments are designed to obtain measurements from 0.5 – 30 keV at a nominal time cadence of 10 s. A description of the MinXSS instruments, performance capabilities, and relation to the *Geostationary Operational Environmental Satellite* (GOES) 0.1 – 0.8 nm flux is given in this article. Early MinXSS results demonstrate the capability of measuring variations of the solar spectral soft X-ray (SXR) flux between 0.8 – 12 keV from at least GOES A5–M5 ($5 \times 10^{-8}\,\mbox{--}\,5 \times10^{-5}~\mbox{W}\,\mbox{m}^{-2}$) levels and of inferring physical properties (temperature and emission measure) from the MinXSS data alone. Moreover, coronal elemental abundances can be inferred, specifically for Fe, Ca, Si, Mg, S, Ar, and Ni, when the count rate is sufficiently high at each elemental spectral feature. Additionally, temperature response curves and emission measure loci demonstrate the MinXSS sensitivity to plasma emission at different temperatures. MinXSS observations coupled with those from other solar observatories can help address some of the most compelling questions in solar coronal physics. Finally, simultaneous observations by MinXSS and the *Reuven Ramaty High Energy Solar Spectroscopic Imager* (RHESSI) can provide the most spectrally complete soft X-ray solar flare photon flux measurements to date.

## Introduction

The objective of the *Miniature X-ray Solar Spectrometer* (MinXSS) CubeSats is to explore the highly variable solar soft X-ray (SXR) spectral distribution and reveal its impact on Earth’s ionosphere and thermosphere. The MinXSS X-ray instruments consist of a spectrometer, called X123, with a nominal 0.15 keV full-width at half-maximum (FWHM) resolution at 5.9 keV and a broadband X-ray photometer, called XP. Both instruments are designed to obtain measurements from 0.5 – 30 keV at a nominal time cadence of 10 s. Solar soft X-rays are strongly absorbed by Earth’s atmosphere in the E-region at an altitude of about ${\approx}\,50\,\mbox{--}\,80~\mbox{km}$. This energy input can strongly affect the energetics and dynamics of the ionosphere and thermosphere. These solar measurements can also be used to directly investigate the properties of the solar corona, which is dominated by magnetic field dynamics, resulting in tenuous, high-temperature plasma of over 1 MK. The primary heating source or the relative contributions of the many components to coronal heating are still in question. While MinXSS data alone cannot address the root of this question, the spectrally resolved measurements from MinXSS combined with other solar observations can yield critical information on this and other compelling questions in solar physics. MinXSS is not the first spectrometer to conduct this type of measurements, but it has unique new capabilities that can be advantageous over previous full-Sun flux (irradiance) measurements from spatially integrating spectrometers.

Currently, there are no solar instruments continuously conducting spectrally resolved soft X-ray measurements over a relatively large energy range. Fairly recent spectrally resolved, spatially integrated measurements have been conducted by the *Solar Photometer in X-rays* (SphinX: Gburek *et al.*, 2013) onboard the *Complex Orbital Observations Near-Earth of Activity of the Sun-Photon* (CORONAS-Photon) satellite and the *Solar Assembly for X-rays* (SAX: Schlemm *et al.*, 2007) onboard the *Mercury Surface, Space Environment, Geochemistry, and Ranging* (MESSENGER) satellite. SphinX conducted solar measurements over a time frame of roughly nine months in 2009 during a time of very low solar X-ray flux levels, including the lowest solar X-ray levels ever recorded. The SphinX designed spectral coverage was ${\approx}\,1\,\mbox{--}\,15~\mbox{keV}$ at a nominal 0.4 keV spectral resolution. MESSENGER/SAX performed solar measurements primarily at an orbit around Mercury from 1 – 10 keV at a nominal spectral resolution of 0.6 keV and has measured numerous solar flares (Dennis *et al.*, 2015) from March 2011 to April 2015. Like the MinXSS solar X-ray measurements, both of these spectrometers generated spatially integrated spectra over the instrument fields of view (FOV). MinXSS is designed to greatly improve upon these measurements and enhance the ability of determining emission line features with an improved spectral resolution (nominally ${\approx}\,0.15~\mbox{keV}$ at 5.9 keV, owing to detector architecture and electronics), a lower energy threshold ($E_{\mathrm{ph}} \gtrsim 0.8~\mbox{keV}$, owing to a slightly thinner Be window for MinXSS-2), and by providing near-continuous dedicated solar measurements over a period of about six years (MinXSS-1 is a one-year mission, and MinXSS-2 is a five-year mission) during the declining phase of Solar Cycle 24, throughout solar minimum, and into the rising phase of Solar Cycle 25. MinXSS-1 was deployed from the *International Space Station* on 16 May 2016 and performed solar measurements until re-entry into Earth’s atmosphere on 6 May 2017. MinXSS-2 is scheduled to launch no earlier than (NET) June 2018.

### Current Solar Soft X-Ray Measurements

The aforementioned solar soft X-ray measurements lack direct spatial information. Combining spatially integrated, spectrally resolved measurements of MinXSS with data from other solar X-ray observatories can provide information on solar conditions. The *X-ray Telescope* (XRT: Kosugi *et al.*, 2007; Golub *et al.*, 2007) onboard *Hinode* uses filters to create spectrally separated images of the soft X-ray intensity, but lacks fine spectral knowledge. The *Soft X-ray Imager* (SXI: Hill *et al.*, 2005) onboard the *Geostationary Operational Environmental Satellite* (GOES) exhibits similar qualitative spectral capabilities as XRT, but also suffers from the same issue of spectrally convolved images. The *X-ray Sensor* (XRS: Garcia, 1994) onboard GOES conducts spectrally and spatially integrated measurements in two bands (0.1 – 0.8 nm and 0.05 – 0.4 nm). A ratio of these two bands can yield an isothermal approximation to the coronal plasma temperature at the time of measurement (White, Thomas, and Schwartz, 2005). The *Reuven Ramaty High Energy Solar Spectroscopic Imager* (RHESSI: Lin *et al.*, 2002) has provided spectral and spatial information using a Fourier imaging technique. The primary spectral coverage of RHESSI extends from 6 keV – 17 MeV, with systematics-limited sensitivity below 6 keV. The best spectral resolution for RHESSI in the 3 – 100 keV bandpass is ${\approx}\,1~\mbox{keV}$ FWHM coupled with a 2.3 arcsecond FWHM spatial resolution. Additionally, the astronomy-based *Nuclear Spectroscopic Telescope Array* (NuSTAR: Harrison *et al.*, 2013) satellite has performed a series of solar measurements that has been summarized in Grefenstette *et al.* (2016) and Hannah *et al.* (2016). The NuSTAR nominal 0.4 keV FWHM spectral resolution can produce spectral images with ${\approx}\,18~\mbox{arcsecond}$ spatial resolution over its ${\approx}\,11~\mbox{arcminute}$ FOV.

### MinXSS Science Goals

MinXSS data combined with the soft X-ray instrument data mentioned earlier can be used in conjunction with other UV and visible space observatories such as the *Solar Dynamics Observatory* (SDO: Pesnell, Thompson, and Chamberlin, 2012), the *Hinode/EUV Imaging Spectrograph* (EIS: Culhane *et al.*, 2007) and the *Hinode/Solar Optical Telescope* (SOT: Tsuneta *et al.*, 2008), and the *Interface Region Imaging Spectrometer* (IRIS: De Pontieu *et al.*, 2014), to mention a few, to address pertinent science questions about the solar atmosphere. Specifically, the high temperature, low density, and magnetic conditions of the corona have elicited keen interest for decades since the observations of “coronium” lines in the solar spectrum (Rayet, 1868; Seechi, 1875; Golub and Pasachoff, 2010). Better understanding of the solar soft X-ray energy distribution allows for improved inferences of plasma conditions present during various stages of the solar cycle and during solar flares. In addition, to help understand the solar soft X-ray influence on Earth’s ionosphere and thermosphere, a few main solar science questions and tasks that MinXSS data will help address are the following:

- What is the solar soft X-ray energy distribution as a function of solar cycle phase (at least the falling and rising phases) for the following components:
  - flares
  - active regions (AR)
  - the quiescent Sun (QS)?
- What is the AR variation in temperature, density, emission measure, chemical composition, and magnetic complexity as a function of its age and solar cycle phases?
- How are processes different between eruptive and non-eruptive compact flares?
- What is the soft X-ray spectral connection to magnetic complexity in the solar atmosphere?

In order to effectively include MinXSS data in any solar analysis, it is necessary to understand the performance capabilities of the MinXSS X-ray instruments. This article describes the basic instrument characteristics and will be a reference for scientists interested in utilizing MinXSS. The remainder of this article includes basic descriptions of the MinXSS CubeSat mission in Section 2, an overview of the instruments in Section 3, capabilities of these instruments in Section 4, followed by examples of MinXSS-1 measurements from low solar levels (GOES A5) to an M5 flare and plasma inferences in Section 5. Additional references for MinXSS include an overview of the MinXSS CubeSat and its subsystems by Mason *et al.* (2016), pre-flight calibration results by Moore *et al.* (2016), and early mission results by Woods *et al.* (2017).

## The MinXSS CubeSat Missions

**Figure 1:**
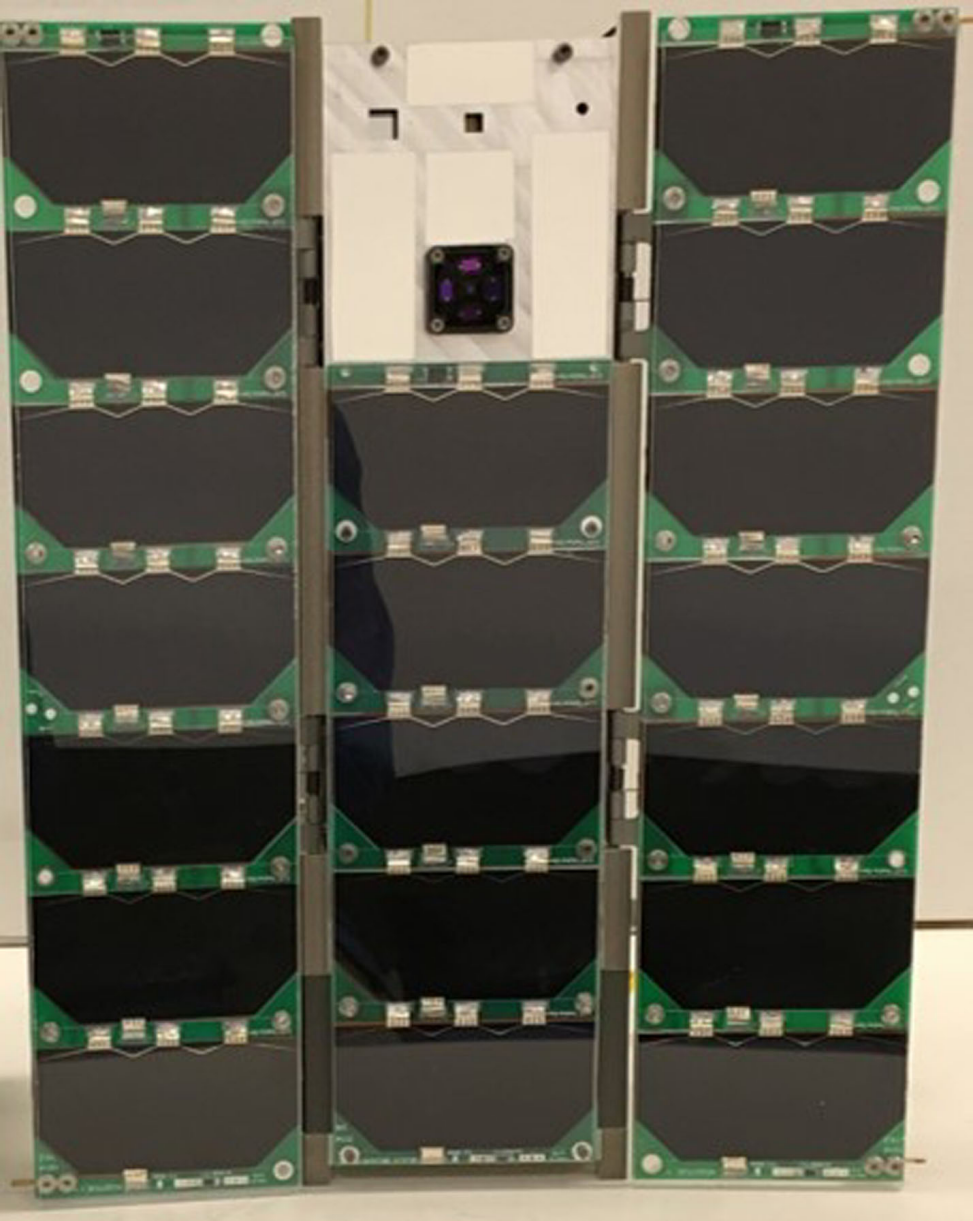
Picture of one of the twin *Miniature X-ray Solar Spectrometer* (MinXSS) 3U CubeSats. The CubeSat is oriented so that the solar panels and instrument apertures are facing the viewer (desired Sun-facing side on-orbit). The MinXSS CubeSats are designed to measure the solar X-ray flux from 0.5 – 30 keV using the X-ray photometer (XP) for spectrally integrated measurements across the entire energy band and the X123 spectrometer for energy-resolved photon-counting measurements. The X123 spectrometer has a nominal spectral resolution of 0.15 keV FWHM. MinXSS-1 was deployed from the *International Space Station* on 16 May 2016 with an initial LEO altitude of ${\approx}\,402~\mbox{km}$ for an anticipated mission lifetime of ${\approx}\,12~\mbox{months}$, depending on solar activity. MinXSS-2 is to be launched to a Sun-synchronous orbit of ${\approx}\,500~\mbox{km}$ NET June 2018 for an anticipated five-year mission.

**Figure 2:**
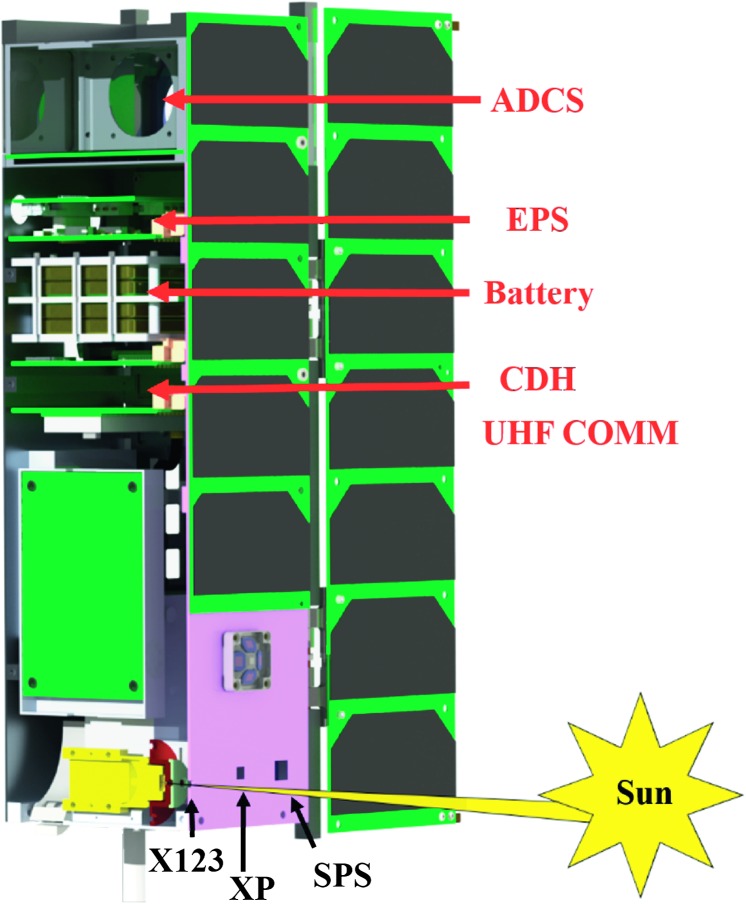
Cut-out of the MinXSS CubeSat to demonstrate the optical path to the Sun (not to scale) through the FOV-limiting aperture and housing for the X123 spectrometer. The X123, XP, SPS apertures, and other subsystem locations are labeled for clarity.

The development and testing of the MinXSS CubeSats involved extensive graduate student involvement in collaboration with the University of Colorado Laboratory for Atmospheric and Space Physics (LASP) in Boulder, and the Aerospace Engineering Sciences Department, with assistance from professors and professionals. The first of the twin satellites, MinXSS-1, was ferried to the *International Space Station* (ISS) from the Kennedy Space Center on 6 December 2015. MinXSS-1 was deployed from the ISS on 16 May 2016 to a low Earth orbit (LEO) with an initial altitude of ${\approx}\,402~\mbox{km}$. MinXSS-1 commenced science operations on 9 June 2016. The second CubeSat, MinXSS-2, is scheduled to launch to a Sun-synchronous orbit NET June 2018. Figure 1 is a picture of the MinXSS-1 3U CubeSat, noting that one unit (1U) is $10~\mbox{cm} \times 10~\mbox{cm} \times 11.35~\mbox{cm}$ in size. Even though they are small relative to “traditional” solar observing satellites, the MinXSS CubeSats are fully functioning satellites. They include triple junction GaAs solar cells from AzurSpace, Li-polymer batteries, an electrical power system (EPS), an attitude and determination control system (ADCS) supplied by Blue Canyon Technologies, a tape measure for a radio antenna, a Li-1 radio for communication, Command and Data Handling (CDH) microcontroller, and science instruments. The positions of these subsystems in the MinXSS spacecraft are shown in Figure 2. An overview of these subsystems is described in Mason *et al.* (2016). MinXSS-2 is an augmented version of MinXSS-1 with planned improvements in hardware, software, and the implementations of lessons learned from the MinXSS-1 mission. Scientifically, one of the most important upgrades of MinXSS-2 is a newer version of the X-ray spectrometer. The MinXSS-1 instruments are listed in Table 1 and consist of a visible-light Sun position sensor (SPS), an X-ray photometer (XP), and an X-ray spectrometer called X123. Nominally, the MinXSS science data are composed of 10-second integrations that can be decreased to 3 seconds or increased to 1 min, depending on the scientific objectives. The next section describes the full set of MinXSS instruments and their capabilities.

**Table 1:** MinXSS satellite launch, orbit, and mission lifetimes. A few instrument properties are also listed.

Satellite	MinXSS-1			MinXSS-2		
Orbit insertion date	16 May 2016			NET^a^ Jun. 2018		
Anticipated mission lifetime	${\approx}\,1~\mbox{year}$			${\approx}\,5~\mbox{years}$		
Initial orbit altitude (km)	${\approx}\,400~\mbox{km}$			${\approx}\,500~\mbox{km}$		
Instrument	SPS	XP	X123	SPS	XP	X123
Si detector depletion depth (μm)	55	55	500	55	55	500
Aperture area (cm^2^)	4.0 × 10^−2^	2.0 × 10^−1^	2.5 × 10^−4^	4.0 × 10^−2^	2.0 × 10^−1^	2.5 × 10^−4^
Window type-material	ND7^b^	Be	Be	ND7^c^	Be	Be + Zn
Window thickness (μm – uncertainty)	–	19.0 (0.1)	24.5 (0.6)	–	18.0 (0.1)	11.2 (0.3)+0.1 (0.1)
Full field of view (FOV, ^∘^)	8	8	4	8	8	4

^a^NET = Not earlier than.

^b^Neutral density filter – 10^7^.

^c^Neutral density filter – 10^7^.

## MinXSS Instrument Description

### Sun Position Sensor (SPS)

The SPS is a visible-light-sensitive quad silicon-photodiode (Si-photodiode) arrangement behind an ND7 (neutral density filter with an attenuation factor of $10^{7}$). The Si-photodiodes have a depletion depth of 55 μm. The SPS square aperture is $2 \times 2~\mbox{mm}$, and the layout is discussed in the Appendix and in Figure 16. The relative solar illumination on each of the four diodes is used to calculate the solar position within the MinXSS X-ray instrument FOVs. The absolute position knowledge is accurate to within 10 arcseconds and the pointing is controlled to a precision of 10 arcseconds, limited by the capabilities of the Blue Canyon XACT ADCS system.

### X-Ray Photometer (XP)

The LASP-designed XP unit consists of a Si-photodiode (depletion depth of 55 μm) with a beryllium (Be) window 19.0 μm thick for MinXSS-1 and 18.0 μm thick for MinXSS-2 to attenuate visible-light contamination. The XP aperture is 5 mm in diameter. The main purpose of XP is to serve as a consistency check to the detected photon flux and linearity of the X-ray spectrometer X123, assessing long-term degradation trends, and comparison to the GOES/XRS photometers. The Si-photodiodes in XP and SPS have been flown numerous times, such as on the SDO (Woods *et al.*, 2012), are known to be stable in space, and have linear response over the full range of solar flux levels after corrections to various influences. The XP photodiode response can have gain and dark current variations as a result of thermal fluctuations, and can possibly suffer noise from sources internal to the MinXSS spacecraft (electromagnetic field, radio transmission, microphonics, *etc.*), which must be corrected for. After gain and dark current corrections, the XP product returns one spectrally integrated value for the solar X-ray contribution over its expected spectral response from ${\approx}\,0.5\,\mbox{--}\,30~\mbox{keV}$. This type of information is valuable, but of similar class to the GOES/XRS. The spectral information provided by the X-ray spectrometer, X123, can greatly enhance the interpretation of the XP data.

### X-Ray Spectrometer (X123)

The MinXSS main scientific instrument, X123, is an X-ray spectrometer purchased from Amptek (http://amptek.com/). The data returned from X123 will be the most important data product that MinXSS will provide to the scientific community. X123 is composed of a silicon drift detector (SDD) with a 500 μm thick Si depletion depth behind a Be window 24.5 μm thick for MinXSS-1 and 11.2 μm thick for MinXSS-2. From pre-flight calibrations, there appears to be Zn contaminant in the MinXSS-2 Be filter with an approximate thickness of 0.1 μm. The X123 dead layer is comprised mostly of SiO and has a nominal thickness around 0.15 μm. We did not measure the dead-layer thickness ourselves, but include the nominal value into our detector modeling. This thickness of SiO does not strongly impact the detector high-energy efficiency, but could contribute to the low-energy ($<1~\mbox{keV}$) efficiency.

Amptek provides the X123 electronics for power regulation, X-ray photon energy detection, and embedded software for reading the spectrometer output. Thus, in addition to X-ray characterization, the main task remaining is to integrate the X123 output to the MinXSS CDH processor that compresses the data and formats it into data packets. The power draw from X123 is nominally ${\approx}\,2.8~\mbox{W}$ during normal operations and at maximum ${\approx}5.0~\mbox{W}$ (normally at turn-on). The X123 spectrometer is actively cooled with a thermoelectric cooler (also provided by Amptek) to ${\approx}\,224~\mbox{K}$. The cooling is necessary to minimize thermal noise, which will contribute to the lower energy bins ($E_{\mathrm{bin}} \leq 1.0~\mbox{keV}$). The X123 sensor resides inside a stainless steel housing behind a tungsten FOV-limiting pinhole aperture with a ${\approx}\,0.18~\mbox{mm}$ diameter to protect from energetic particles and hard X-rays.

### Detector Operation

The X123 SDD, XP, and SPS operation relies, in principle, on electron-hole pair generation in the Si lattice by the incident photon flux. Photons and any other energy sources (these contribute to the noise) that have more than the Si electron-hole pair generation energy ($E_{\text{e-h}} \gtrapprox 3.65~\mbox{eV}$) can create a number of electron-hole pairs proportional to the energy of the incident photon. For the SPS and XP detectors, the liberated charges contribute to a current that is measured, amplified, and converted to a digital unit. The difference for the X123 spectrometer is that the electrons generated by this energy deposition process drift toward the readout anode, and the resulting charge is integrated on a capacitor within the detector rise time (peaking time). These energy impulses are deemed events. The preamplifier, which consists of a field-effect transistor (JFET for MinXSS-1 and MOSFET for MinXSS-2) that amplifies the signal, converts it into a voltage, and this voltage ramp is shaped to a trapezoid. Each of these shaped signals are used to discern the energy deposited in the active detector volume by the incident photon event. The performance capabilities of the X-ray instruments are discussed in the next section.

## MinXSS X-Ray Instrument Capabilities

The X123 spectrometer was characterized using radioactive laboratory sources for spectral resolution, optimization of electronic settings, and determining the gain and energy offset. SPS, XP and X123 FOV, XP and X123 window thicknesses, spectral efficiency, and linearity were determined from the National Institute for Standards and Technology (NIST) Synchrotron Ultraviolet Radiation Facility (SURF) measurements. MinXSS/X123 spectrometer basic performance properties and characterization methodology are described in Moore *et al.* (2016). Accurate knowledge of the electron beam current, electron beam energy, magnetic field strength, and source distance allows for the precise calculation of the synchrotron light spectral intensity to enable calibration of the X123 and XP responsivities with about 10% accuracy (Moore *et al.*, 2016).

### Field-of-View Sensitivity

The MinXSS X-ray instruments integrate the incident radiation across their FOVs to create their respective signals. While XP and X123 do not have spatial resolution *per se*, they do possess a sensitivity to light intensity across their respective FOVs. XP nominally has an angular response across an $8^{\circ}$ FOV and X123 has an FOV of $4^{\circ}$. An example of the alpha–beta ($\alpha$–$\beta$) coordinate system used for the SURF calibrations is given in the SPS image in Figure 16. An extended source such as the Sun viewed from Earth will have an angular size of roughly $0.5^{\circ}$, but the response can vary strongly depending on the actual X-ray coronal structure during observations. This is well within the FOV of XP and X123. For the most accurate photometric measurements from MinXSS, it is important to correct for the solar disk position within the MinXSS FOV.

**Figure 3:**
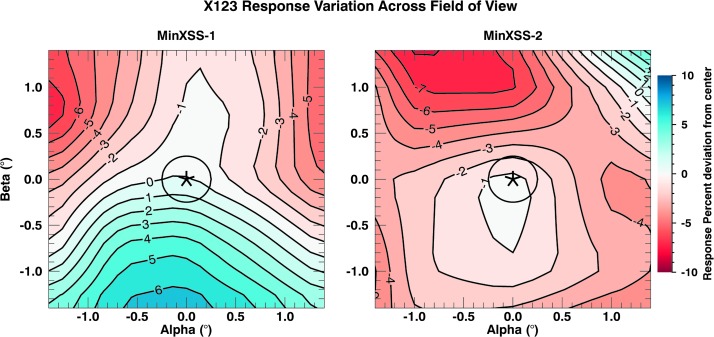
X123 FOV sensitivity maps constructed from pre-launch data at the National Institute for Standards and Technology (NIST) Synchrotron Ultraviolet Radiation Facility (SURF) for each CubeSat (*left*: MinXSS-1, and *right*: MinXSS-2). The maps displayed are the MinXSS spectral response convolved with the apparent visible-light solar disk. The *asterisk* denotes the center of the spectrometer FOV, which is mechanically aligned to the boresight of the spacecraft. The *black circle* represents the size of the visible-light solar disk in the FOV. The contours and color map signify the percent difference in the X123 response from the center (*asterisk*), which is a few percent in magnitude.

Figure 3 shows FOV sensitivity maps of MinXSS-1 and MinXSS-2 X123 units. The maps are a $1.4^{\circ}\times1.4^{\circ}$ subset, centered on the FOV of X123. The XP map yields similar FOV sensitivities. The values are the raw NIST SURF determined MinXSS response maps, convolved with the visible-light solar disk, and expressed as a percent difference in the total signal summed over all energies at that position with respect to the center, to estimate the X-ray detection variation across the FOV. This pre-flight map serves as our baseline until on-orbit maps are created for comparison. The absolute variation across the centered $1.4^{\circ}\times1.4^{\circ}$ FOV is around 8%. The MinXSS ADCS unit (XACT from Blue Canyon Technologies) has proven to keep the Sun within $0.3^{\circ }$ of the center of the MinXSS FOV with a precision of 10 arcseconds (${\approx}\,0.028^{\circ}$). Thus, the inner $1^{\circ}$ diameter annular region is the most important for MinXSS on-orbit performance, and the absolute variation within this region is no larger than about 5%. We note that only mechanical alignment was implemented between the XACT ADCS unit and the instruments.

### Spectral Resolution

The X123 spectral resolution, energy bin gain, and offset have been estimated using radioactive line sources of ^55^Fe for the ${\approx}\,5.90$ and ${\approx}\,6.49~\mbox{keV}$ line complexes, and ^241^Am for the ${\approx}\,11.87$, ${\approx}\,13.95$, and ${\approx}\,17.75~\mbox{keV}$ lines. The fit values for both the MinXSS-1/X123 gain and MinXSS-2/X123 gain are 0.0297 keV/bin and are consistent with the nominal bin width (0.03 keV). The offset is dependent on the electrical grounding conditions at the time of operation because the pulse-height analysis is computed on top of a baseline voltage, which can drift. Thus, the X123 energy offset in the laboratory at LASP for radioactive source measurements, at NIST SURF, and on-orbit may not be exactly the same. Nominally, the value for MinXSS-1 energy bin offset is around $-0.076~\mbox{keV}$, and the MinXSS-2 energy bin offset is close to $-0.265~\mbox{keV}$.

The X123 has a customizable peaking time, which dictates how long the photon-liberated electron charge cloud is integrated on the readout capacitor. The longer the peaking time, the better the spectral resolution. MinXSS-1 has a fixed peaking time of 4.8 μs, but we have tested the MinXSS-2/X123 detector for various peaking times between 0.6 – 9.6 μs. We will operate the MinXSS-2/X123 for peaking times between 1.2 – 4.8 μs because of the tradeoff between spectral resolution and effective maximum count rate that can be recorded before photon pile-up begins to occur. We only show data for these 1.2 – 4.8 μs peaking times in this article. The longer the peaking time, the lower the maximum count rate that can be accurately measured. Details on the maximum count rate are discussed in Section 4.6.

**Figure 4:**
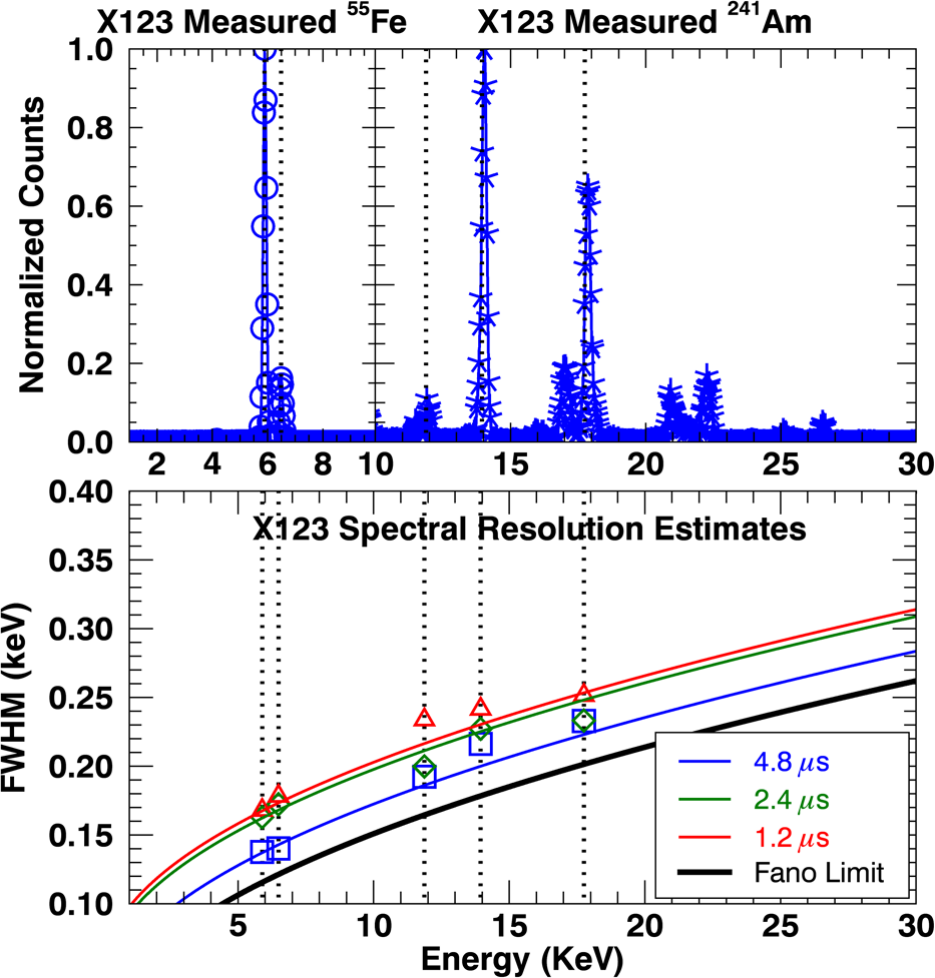
Example plot of MinXSS-2/X123 spectral resolution estimates *vs*. photon energy using radioactive X-ray sources. The *top split-plot* shows the normalized counts from the ^55^Fe source from 1 – 10 keV and the ^241^Am source from 10 – 30 keV. The ^55^Fe ${\approx}\,5.90$ and ${\approx}\,6.49~\mbox{keV}$ line complexes are easily detected. The ^241^Am ${\approx}\,11.87$, ${\approx}\,13.95$, and ${\approx}\,17.75~\mbox{keV}$ lines are used for spectral resolution estimates. The *vertical dotted line* emphasizes which spectral lines were used for FWHM resolution estimates for three different spectrometer peaking times. Longer peaking times yield better photon energy resolving power up to the combined electronic and Fano limit. The Fano limit is the intrinsic statistical limit of bulk Si semiconductor material to resolve energy differences, and is overplotted with the black solid line. The *colored lines* are the Fano limit (from Fano noise) with an estimated electronic noise contribution from the ^55^Fe measurements. These estimates depicted by the color lines are used to extrapolate the spectral resolution to higher photon energies. The extrapolation to lower energies is not expected to adhere to these lines because of other noise sources (microphonics, thermal noise, and other uncharacterized sources).

Figure 4 shows example MinXSS-2/X123 ^55^Fe measurements from 1 – 10 keV and ^241^Am measurements from 10 – 30 keV. The subset of lines used to assess the spectral resolution at specific energies are signified by the vertical dotted lines. An expanded plot of the ^55^Fe for the ${\approx}\,5.90$ and ${\approx}\,6.49~\mbox{keV}$ line complexes are presented in Moore *et al.* (2016). The nominal spectral resolution near 5.9 keV was confirmed for the respective peaking times. MinXSS-1/X123 detector resolution measurements were not performed in the same way as for the MinXSS-2 detector. Thus, we currently estimate the on-orbit resolution to be ${\approx}\,0.24~\mbox{keV}$, which is much broader than the nominal 0.15 keV spectral resolution at 5.9 keV for a 4.8 μs peaking time. We are currently assessing the reasons for a degraded on-orbit resolution. MinXSS-2 has an improved version of the X123 spectrometer, the X123 Fast SDD. The X123 Fast SDD has an improved preamplifier, a MOSFET transistor instead of a JFET for the MinXSS-1 version. This has a lower effective capacitance and lower noise and results in an improved spectral resolution for the same peaking times as the older version. An advantage is the increased maximum count rate, which we discuss in Section 4.6. The spectral resolution for MinXSS-2 at 5.9 keV is 0.137 keV for 4.8 μs, 0.162 keV for 2.4 μs, and 0.168 keV for 1.2 μs.

The resolving power at X-ray energies for Si-based detectors is limited by Fano noise (Knoll, 2010). Fano noise is the intrinsic statistical variation in the electron-hole pair-generation rate *per* event and is represented by the solid black line in Figure 4. The Fano factor ($F$) is essentially the ratio between the observed variance to the expected Poisson variance, and for silicon, this is around 0.1. The Fano noise in FWHM units is $\sigma_{\mathrm{Fano}} = 2.35\times\sqrt{\frac{F\times E_{\mathrm{ph}}}{E_{\text{e-h}}}}$, where $E_{\mathrm{ph}}$ is the photon energy and $E_{\text{e-h}}$ is the electron-hole pair generation. The electronic noise contribution was estimated from the ^55^Fe for the ${\approx}\,5.90$ and ${\approx}\,6.49~\mbox{keV}$ data and used to scale up the Fano noise to provide an estimate of the spectral resolution at the higher energies. The extrapolation to lower energies is not expected to adhere to these lines because of other noise sources (microphonics, thermal noise, and other uncharacterized sources).

### Spectral Efficiency and Effective Area

The XP 55 μm and X123 500 μm SDD thick depletion depths for Si-based X-ray detectors dictate the high-energy cut-off efficiency. The window material and composition affect the high- and low-energy efficiency. For Be-based windows, only the lower energy photons are attenuated, and thus the Be window thickness dictates the low-energy photon response. Noise contributions, primarily electronic noise, limit the XP signal fidelity and the X123 lower energy contribution. The MinXSS instrument spectral efficiency was determined through a series of measurements conducted at NIST SURF, and the details are listed in Moore *et al.* (2016). The SURF photon spectrum is known to within 10% near 1 keV and produces statistically significant counts up to about 3 keV. Model uncertainties in the fitted detector window thicknesses, depletion depth thicknesses, and atomic coefficients yield uncertainties near 20% for the higher energies. Since the SURF spectrum is a continuum, it is not very useful for the spectral resolution estimates, such as those discussed in Section 4.2, but the absolute synchrotron photon spectral distribution can be used to determine the detection efficiency of the MinXSS X-ray instruments.

The MinXSS/XP signal can be calculated from the following expression in Equation 1, where $C_{\mathrm{XP}}$ is the XP count rate with units of DN *per* second, with DN the data number, which is converted from the femtoCoulomb (fC) signal by the XP gain, $G_{\mathrm{XP}}$. The bracketed term is the femtoAmps (fA) generated from the detected photon flux, from the source of photons, $S(E_{\mathrm{ph}}, \Omega)$, with photon energy $E_{\mathrm{ph}}$. Then,

1 $$ C_{\mathrm{XP}} = G_{\mathrm{XP}} \int_{0}^{\infty } \biggl[ \int^{\Omega_{\odot}} S(E_{\mathrm{ph}}, \Omega) A_{\mathrm{XP}} R_{\mathrm{XP}}(E_{\mathrm{ph}}, \Omega) \,\mathrm{d}\Omega \biggr] \,\mathrm{d}E_{\mathrm{ph}}. $$

In the case of the Sun, there is a distribution of photons as a function of X-ray energy, and a function of position in the extended corona. The Sun appears as an extended object, and its position can vary within the MinXSS FOV. The physical extent of the X-ray emission from the Sun is encompassed in the solid angle, $\Omega$, which is not necessarily the simple conversion of $\pi \sin^{2}(\theta_{\odot}) \approx\pi (\frac{R\odot}{1~\text{AU}} )^{2} \approx6.8\times10^{-5}~\mbox{sr}$ because the X-ray emission is not confined to the visible-light solar disk. An example of the distribution of X-ray emission that MinXSS can detect is illustrated in the *Hinode*/XRT images in Figure 11. Thus, $S(E_{\mathrm{ph}}, \Omega)$ is an intensity or radiance, in units of photons $\mbox{s}^{-1}\,\mbox{keV}^{-1}\,\mbox{cm}^{-2}\,\mbox{sr}^{-1}$ in this formulation. $A_{\mathrm{XP}}$ is the XP aperture geometric area in units of $\mbox{cm}^{2}$. The XP detector response, $R_{\mathrm{XP}}(E_{\mathrm{ph}}, \Omega)$, includes the XP detector efficiency, the conversion from photon energy into fC, $\epsilon_{\mathrm{ph}}$, and the FOV sensitivity, which is encompassed in the $\Omega$ dependence. In theory, the integral of the bracketed term can include photon energies from 0 to $\infty$ incident on the XP area, but the actual high- and low-energy limits are set by the XP detector response.

**Figure 5:**
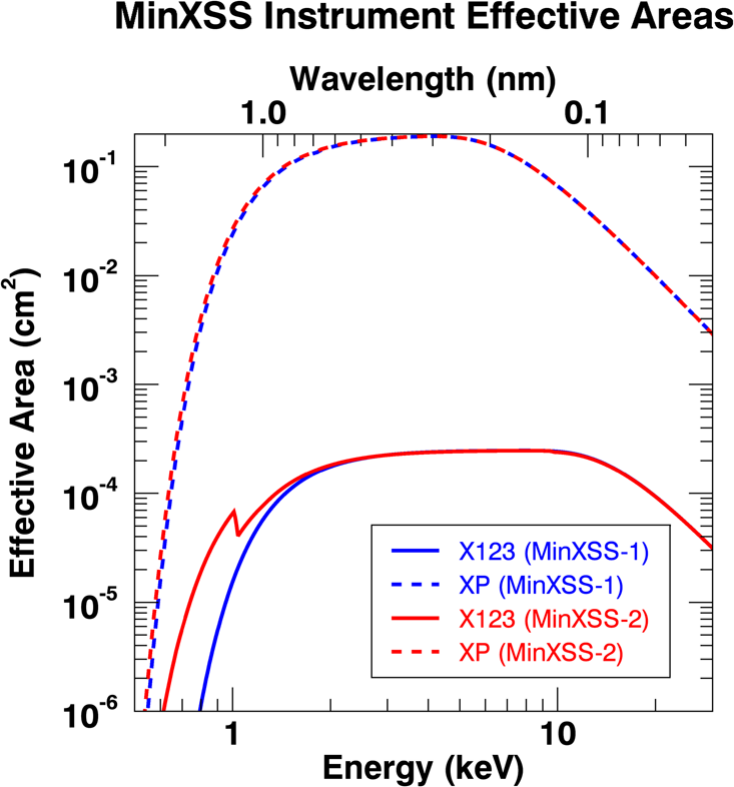
MinXSS-1 and MinXSS-2 X-ray instrument effective area *vs*. photon energy. The main difference between the XP (*dashed line*) and X123 (*solid line*) is due to the geometric area of their respective apertures. The XP aperture diameter is ${\approx}\,5~\mbox{mm}$, while the X123 pinhole diameter is ${\approx}\,0.18~\mbox{mm}$. MinXSS-2 has an undesigned Zn contribution to the Be window, which results in an edge in the response near 1 keV.

The combination of the XP detector response and geometric area constitutes an “effective area”. The XP MinXSS-1 and MinXSS-2 effective area curves are displayed in Figure 5. The main XP photon response lies between 0.5 – 30 keV, with the actual count contribution depending on the solar X-ray spectrum, $S(E_{\mathrm{ph}}, \Omega )$, during observations. The MinXSS-1 and MinXSS-2 XP devices both have similar responses because their Be windows are of similar thickness (19.0 μm for MinXSS-1 and 18.0 μm for MinXSS-2).

In a similar fashion to the XP calculation, the X123 count rate *per* energy bin, denoted as $j$, is $(C_{\text{X123}})_{\text{bin},j}$, in units of counts $\mbox{s}^{-1}$, can be calculated by Equations 2 and 3,

2 $$\begin{aligned} (C_{\text{X123}} )_{\text{bin},j} =& \int_{E_{\mathrm{min},j}}^{E_{\mathrm{max},j}} \bigl[ \Upsilon(E_{\mathrm{det}}) \bigr] \,\mathrm{d}E_{\mathrm{det}} , \end{aligned}$$

3 $$\begin{aligned} \Upsilon(E_{\mathrm{det}}) =& \int_{0}^{\infty} \int^{\Omega_{\odot}} S(E_{\mathrm{ph}}, \Omega) A_{\text{X123}} \overline{\Re}_{\text{X123}} (E_{\mathrm{ph}}, \Omega, E_{\mathrm{det}}) \,\mathrm{d}\Omega \,\mathrm{d}E_{\mathrm{ph}} , \end{aligned}$$

where $A_{\text{X123}}$ is the X123 aperture geometric area. The main difference between XP and X123 in terms of the signal calculation is the spectral binning of the data and the response function, $\overline{\Re}_{\text{X123}} (E_{\mathrm{ph}}, \Omega, E_{\mathrm{det}})$, which is the photon ⇔ detected energy bin redistribution function and includes the FOV dependence through $\Omega$. The function $\overline {\Re}_{\text{X123}} (E_{\mathrm{ph}}, \Omega, E_{\mathrm{det}})$ maps $E_{\mathrm{ph}}$ to $E_{\mathrm{det}}$ for forward modeling and *vice versa*, for inverting detected counts to create an incident photon flux estimate. Thus, the bracketed term in Equation 2 is only a function of $\mathrm{d}E_{\mathrm{det}}$ because of the operation of $\overline{\Re}_{\text{X123}} (E_{\mathrm{ph}}, \Omega, E_{\mathrm{det}})$ on the source intensity $S(E_{\mathrm{ph}}, \Omega )$. Hence, the final integral over the energy of the individual energy bin limits, $E_{\mathrm{min},j}$ and $E_{\mathrm{max},j}$, to obtain a final count rate value in energy bin $j$. Again, in theory, the integral over $E_{\mathrm{ph}}$ in the bracket is over all photon energies, but in reality, this is limited to the X123 spectral efficiency and high and low photon limits.

Similarly, for X123, the combination of the X123 spectral efficiency (the probability that a photon incident on the active area of the detector will be absorbed) and the aperture geometric area can be used to create the effective area curves for MinXSS-1/ and MinXSS-2/X123 in Figure 5. The edge near 1 keV in the MinXSS-2/X123 detector effective area curve is due to a Zn contamination in the Amptek-supplied Be window and has been included in the modeling. The primary spectral range is nominally 0.5 – 30 keV for both MinXSS spacecraft X123 detectors, but it is obvious in Figure 5 that the MinXSS-2 detector has a higher efficiency for the lower photon energies ($E_{\mathrm{ph}} \leq 2~\mbox{keV}$). This is due to the thinner 11.2 μm Be window on the MinXSS-2 detector as compared to the 24.5 μm thick window on MinXSS-1. The choice of the thinner Be window X123 detector being on the second MinXSS was made because MinXSS-2 has an anticipated longer mission of about five years *vs*. the MinXSS-1 expected maximum mission lifetime of about one year. The higher efficiency for lower photon energies aids in extending the low-energy limit of the MinXSS-2/X123 data product. Effective area curves are great for comparing both the spectral and intensity sensitivities between various instruments on different observatories.

### Detector Response Matrix

In reality, the resulting count space is discrete and not continuous after the integration of the bracketed quantity in Equation 2 to create the binned data. Thus, one can interpret the photon-count redistribution function as a detector response matrix (DRM) for the X123 spectrometer, with columns, $k$, that connects the incident photon energy to the rows, $j$, which are the losses recorded in the X123 energy bins. This is indicated in Equation 4, where $\overrightarrow{C}_{\mathbf{j}}$ is the detected count rate spectrum, $\overline{\Re}_{\mathbf{k},\mathbf{j}}$ is the X123 DRM, and $\overrightarrow{S}_{\mathbf{k}}$ is the source intensity,

4 $$ \overrightarrow{C}_{j} = \overline{\Re} _{\mathbf{k},\mathbf{j}} \overrightarrow{S}_{k}. $$

**Figure 6:**
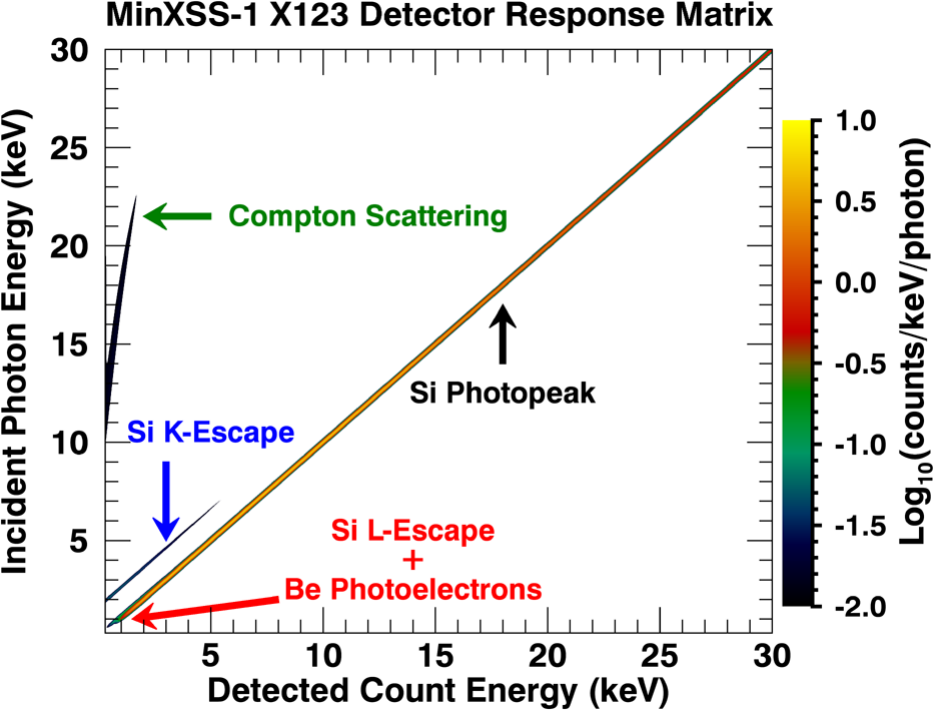
Example of the X123 detector response matrix (DRM), which includes the Si photopeak, Compton scattering, Be-window generated photoelectrons, and Si K and L (2s and 2p) escape processes. The DRM gives the probability that an incident photon of energy $E_{1}$ will deposit energy $E_{2}$ in the detector.

An example of the MinXSS/X123 DRM is displayed in Figure 6. The DRM incorporates the detection efficiency, which is the probability that a photon stopped in the detector by a photoelectric interaction will be recorded. The probability of the full energy being deposited in the detector gives the diagonal response or photopeak efficiency. Additionally, there are loss processes such as fluorescence emission (Knoll, 2010), which result in an event being recorded at a lower energy. These are the off-diagonal terms in the DRM and include loss processes such as Si K and L (2s and 2p) escape. These loss processes result in counts occurring at an energy bin that is the incident photon energy minus the Si edge energy (${\approx}\,1.8~\mbox{keV}$ for K, ${\approx}\,0.15~\mbox{keV}$ for 2s, and ${\approx}\,0.14~\mbox{keV}$ for 2p). These spectral detection shifts occur if the resulting Si escape photon actually leaves the active area of the detector without being absorbed, or else if the escape photon is reabsorbed by the detector, one retains the original photon energy in the detected energy bin.

Another process that we consider is the liberation of photoelectrons in the X123 Be window that can eventually be detected and appear in the spectrum. These events will occur at energies lower than the initial photon distribution that created the photoelectrons through the energy loss processes in migrating to the window surface, the negative bias of the X123 detector front surface and transport through the detector dead layer. The resulting contribution would be a continuum distribution at low energies ($E_{\mathrm{ph}} \leq 1.5~\mbox{keV}$) for typical solar fluxes (peak in photon distribution) and window thicknesses (absorption probability spectrum of the window) on our X123 units. These model contributions are included in the functional form of $\overline{\Re}_{\text{X123}}$ and are also discussed in Moore *et al.* (2016).

Another effect in MinXSS spectra is Compton scattering, but to a much smaller degree than Si escape (both because of the lower probability and the lack of detectable higher energy photons greater than 10 keV). Compton scattering is unlikely to be a major contaminant in the quality of MinXSS spectra because of the small effective area of X123 coupled with the steeply decreasing solar spectra at high energies. Only large X-class flares could produce a large enough signal at photon energies greater than 10 keV to make Compton scattering important, but other issues such as photon pile-up and X123 dead-time will occur before that becomes relevant. We have used a flux linearity test at SURF to quantify when pile-up and detector dead-time will become severe and discuss this in Section 4.6

### Temperature Response

To connect the MinXSS instrument spectral response to the ability of detecting plasma of differing temperatures, one can calculate a temperature response curve. The temperature response curve is the signal expected in an instrument from the plasma photon emission. This response curve is built from many discrete isothermal emission models of differing temperatures and thus is a function of plasma temperature. The temperature response curve is generated using a spectral synthesis model to create an X-ray emission profile from physical parameters (temperature, density, plasma emission measure, elemental abundance, *etc.*) *vs.* photon energy, folding this through the MinXSS instrument response, and totaling the counts over a specified number of energy bins (creating an effective energy bin width for this model) for X123 and the estimated fC for XP. In general, the spectral emission model used is computed for a range of isothermal plasma temperatures. This grid of input temperatures leads to a grid of MinXSS instrument counts *per* isothermal temperature. Equations 5, 6, and 7 show the functional form of the calculation to compute the temperature responses for XP and X123 respectively for completeness:

5 $$\begin{aligned} F(T)_{\mathrm{XP}} =& G_{\mathrm{XP}} \int _{0}^{\infty} \biggl[\int^{\Omega_{\odot}} S(E_{\mathrm{ph}}, \Omega, T) A_{\mathrm{XP}} R_{\mathrm{XP}}(E_{\mathrm{ph}}, \Omega) \,\mathrm{d}\Omega \biggr] \,\mathrm{d}E_{\mathrm{ph}}, \end{aligned}$$

6 $$\begin{aligned} F(T)_{\text{X123}\ \mathrm{bin},j} =& \int _{E_{\mathrm{min},j}}^{E_{\mathrm{max},j}} \bigl[ \Upsilon(E_{\mathrm{det}}, T) \bigr] \,\mathrm{d}E_{\mathrm{det}} , \end{aligned}$$

7 $$\begin{aligned} \Upsilon(E_{\mathrm{det}}, T) =& \int_{0}^{\infty} \int^{\Omega_{\odot}} S(E_{\mathrm{ph}}, \Omega, T) A_{\text{X123}} \overline{\Re}_{\text{X123}} (E_{\mathrm{ph}}, \Omega, E_{\mathrm{det}})\,\mathrm{d}\Omega \,\mathrm{d}E_{\mathrm{ph}} . \end{aligned}$$

**Figure 7:**
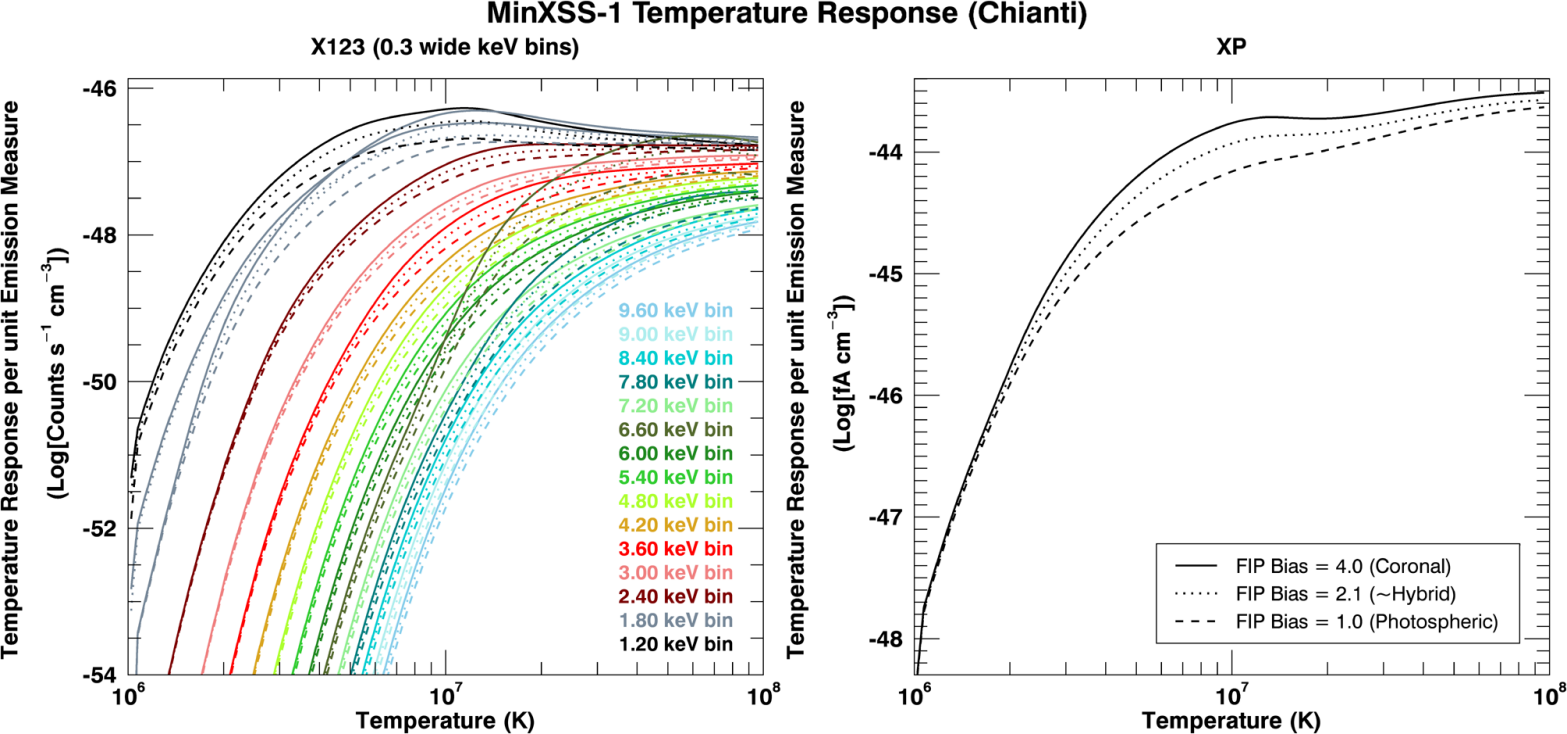
Example of the X123 and XP temperature response functions for a spectrum summed to 0.3 keV wide bins for X123 (ten 0.03 native bins). The temperature response is in volume emission measure units of $\mbox{cm}^{-3}$. The isothermal spectral emission model used to compute the spectral response of the MinXSS instruments *per* plasma temperature is a spectrally extended version of the SolarSoftware (SSW) f_vth function (which uses the Chianti Atomic Database). The temperature response in soft X-rays can vary as a result of differences in the abundance of the low-FIP elements Fe, Mg, Si, Ca, and the mid-FIP element S used in the spectral emission model. Thus, we display the temperature response for traditional “coronal” (Feldman, 1992) (4 times photospheric for the low-FIP elements), “hybrid” (Schmelz *et al.*, 2012) (${\approx}\,2.1$ times photospheric), and one of the latest photospheric (Caffau *et al.*, 2011) abundances. The MinXSS instrument temperature response begins to deviate for different abundances for plasma temperatures greater than 2 MK because of the ions of the low-FIP elements.

An example of the MinXSS/XP and X123 temperature response curves is shown in Figure 7. There are differences in the temperature response function depending on the abundances used in the spectral emission model for the soft X-rays, which are primarily due to the variance in the low first ionization potential (low-FIP) elements Fe, Mg, Si, Ca, and the mid-FIP element S. Elements with a FIP lower than 10 eV have been measured to be overabundant with respect to the high-FIP elements in the solar corona when compared to photospheric values. This has become known as the FIP effect in the Sun. Summaries of the variations in solar abundance are given by Laming (2015) and Schmelz *et al.* (2012). Thus, we calculate the temperature response for a range of abundances and display the abundance values corresponding to “common” reference values in literature. Results displayed are for traditional “coronal” (Feldman, 1992) (4 times photospheric for the low-FIP elements), “hybrid” (Schmelz *et al.*, 2012) (${\approx}\,2.1$ times photospheric), and one of the latest photosphere (Caffau *et al.*, 2011) abundances. The MinXSS instrument temperature response begins to deviate for plasma temperatures greater than 2 MK, primarily because of the ions of the low-FIP elements.

Figure 7 demonstrates the temperature range over which MinXSS/X123 and XP can reliably extract information. X123 and XP inferences are biased toward plasma temperatures greater than 2 MK. The dominant emission for non-large-flaring times is expected to be around 2 – 4 MK. Thus, MinXSS will be able to infer non-large-flaring Sun properties between $1.5\,\mbox{--}\,{\approx}\,4~\mbox{MK}$ with high confidence, but it has limited capabilities for temperatures below 1.5 MK. The temperature response flattens for the X123 lower energy bins (${\leq}\,3~\mbox{keV}$) for temperatures greater than ${\approx}\,4.5~\mbox{MK}$. Owing to this flat nature and with limited significant counts from energy bins greater than 3 keV (which is expected because of the relatively small X123 effective area), X123 can only set upper limits on the emission measure, but cannot definitively constrain the temperature values for plasma hotter than ${\approx}\,5~\mbox{MK}$ during non-large-flaring times. Plasma temperatures above ${\approx}\,5~\mbox{MK}$ are expected for C, M, and X class GOES flares. The higher energy bins (${\geq}\,3~\mbox{keV}$) are mostly sensitive to plasma temperatures greater than ${\approx}\,5~\mbox{MK}$, but need substantial photon flux for statistically significant signals. All these attributes demonstrate that MinXSS has the greatest diagnostic capability for large flares on the Sun.

### Linearity of Response

**Figure 8:**
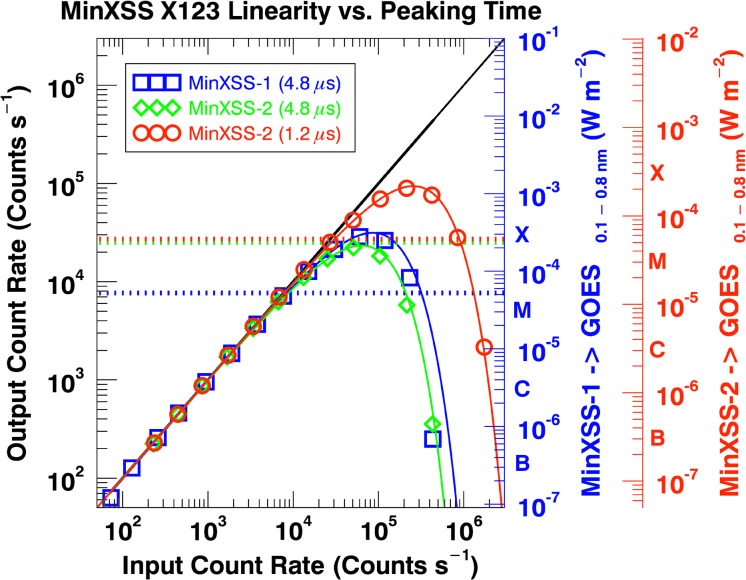
X123 detected (output) count rate for an input (actual) count rate for the MinXSS-1 (*blue symbols*) and MinXSS-2 (*green* and *red symbols*) expected operating peaking times. The *lines* indicate the dead-time model fit. Comparison of a MinXSS-1 observation-based scaling to GOES flux levels (*blue vertical axis*) and model estimations for MinXSS-2 (*red vertical axis*). The *horizontal dotted lines* indicate the count rate where dead-time effects, pile-up effects, and detector paralysis begin to occur. Spectra above these count rates must be heavily processed prior to analysis.

It is desired to have a linear response *vs*. light-source intensity levels for the MinXSS instruments. The linearity of response for XP and X123 was assessed at NIST SURF plus early data from the MinXSS-1 mission, and the data for the former are presented in Figure 8. The count rates discussed here are the measured counts summed over the spectrum during an accumulation divided by the accumulation time. MinXSS data products contain many types of time parameters, but it is best to use the actual measured count rates (counts *per* second, cps) to deduce the severity of dead-time and pile-up effects *vs*. directly using the time parameters in the data sets. The X123 spectral data can be corrected for dead-time losses up until a maximum input count rate for the slow counter, $[{C}_{s}]_{\mathrm{max}}$, which depends on the slow counter peaking time, ${\tau}_{s}$. The slow counter peaking time directly affects the slow counter dead-time, ${\tau}_{ds}$, via the relation ${\tau}_{ds} = B({\tau}_{s} + {\tau}_{\mathrm{flat}})$, where $B$ is a constant (over the mission) *per* detector, and ${\tau}_{\mathrm{flat}}$ is the trapezoidal shaping flat-top time. The maximum input count rate that the slow counter can be corrected for is ${[{C}_{s}]}_{\mathrm{max}} = \frac{1}{{\tau}_{ds}}$. This corresponds to ${\approx}\,85\,000~\mbox{cps}$ for MinXSS-1 (4.8 μs peaking time) and ${\approx}\,255\,000~\mbox{cps}$ for MinXSS-2 (1.2 μs peaking time). Below, we discuss the dead-time correction process with the NIST SURF data and the specific on-orbit correction process.

The MinXSS-1/X123 has a JFET preamplifier, and MinXSS-2 with the X123 Fast SDD unit has a MOSFET preamplifier, allowing for lower noise and improved utility for shorter peaking times. The deduced non-dead-time corrected maximum count rate for MinXSS-1 is ${\approx}\,8\,000~\mbox{cps}$ for its chosen 4.8 μs peaking time. The deduced non-dead-time corrected maximum count rate for MinXSS-2 is ${\approx}\,7\,000~\mbox{cps}$ for the 4.8 μs peaking time and ${\approx}\,27\,000~\mbox{cps}$ for the 1.2 μs peaking time. The latter will be the MinXSS-2 nominal operational peaking time. The horizontal thick dotted lines in Figure 8 display these non-dead-time corrected spectrally integrated maximum count rates. These listed count rate values, ${C}_{s}$, are from the slow counter, which creates the X123 spectrum. The ‘input’ count rate, ${C}_{\mathrm{in}}$, is determined from the measured fast counter count rate, ${C}_{f}$, corrected for fast counter dead-time, ${\tau}_{df}$. The fast counter has a shorter peaking time of 100 ns, ${\tau}_{pf}$, and an effective pair-resolving time of ${\approx}\,120~\mbox{ns}$ (${\tau}_{\mathrm{pair}} = {\tau}_{df} = 120~\mbox{ns}$). The fast channel is used to determine if each event is valid, that is, if the X123 slow channel and hence the digital pulse processor should include it in the spectrum. This helps minimize photon peak pile-up, where more than one photon is absorbed by the detector within the peaking time and the event is recorded as the sum of the photon energies. As a consequence of the much shorter peaking time, the fast channel has a much lower spectral resolution and thus is not the preferred channel for accurate spectra accumulation.

All photon-counting X-ray detectors exhibit some form of count rate loss due to dead-time (Knoll, 2010). At high count rates, losses due to dead-time can become significant, and expressions to approximate the true count rates for X123 are provided by Redus, Huber, and Sperry (2008). The linear black solid line in Figure 8 displays the relation between the input and dead-time corrected fast counter output count rates. The dead-time correction for the fast counter count rate follows the non-paralyzable model, ${C}_{\mathrm{in}} = \frac{{C}_{f}}{(1 - {C}_{f}{\tau }_{df})}$. ${C}_{\mathrm{in}}$ resembles the true input count rate. This expression is accurate to within 5% for true count rates ${\leq}\,500\,000~\mbox{cps}$. For the NIST SURF calibrations, we calculate ${C}_{\mathrm{in}}$ directly from the fast counter and use ${C}_{\mathrm{model}} = {C}_{\mathrm{in}}{e}^{-{C}_{\mathrm{in}}{\tau}_{ds}}$, the paralyzable model, to directly estimate the count rate in the slow counter, ${C}_{s}$. The expression for ${C}_{\mathrm{model}}$ assumes a Poisson arrival probability for photons and registered events. The blue (MinXSS-1, ${\tau}_{s} = 4.8~\upmu \mbox{s}$), green (MinXSS-2, ${\tau}_{s} = 4.8~\upmu \mbox{s}$), and red (MinXSS-2, ${\tau}_{s} = 1.2~\upmu \mbox{s}$) solid lines in Figure 8 are the calculated dead-time suppressed slow counts, ${C}_{\mathrm{model}}$. The corresponding measured slow counts, ${C}_{s}$, are the symbols.

Overall, these predictions agree with the measured data until pile-up effects cause the measured count rate to lie below the calculations. The resultant pile-up effects depend on the shape of the photon flux spectrum, but in general, will be noticeable for input count rate values greater than the peak modeled count rate distribution, ${C}_{\mathrm{model}}$ (the solid lines in Figure 8). This occurs for ${C}_{\mathrm{in}} > \frac{1}{{\tau}_{ds}}$. ${\tau}_{ds}$ will be included in the MinXSS processing software release. Thus, in theory, if one has quality fast and slow counter measurements during an observation, the true count rate can be deduced to within 5% until 500 000 cps. Unfortunately, this NIST SURF procedure for correcting the slow counter for dead-time effects is not directly applicable on-orbit because: 1) the low-energy thresholds for the fast and the slow counter may not be (and currently are not) at exactly the same energy, and 2) on-orbit, the MinXSS-1 fast counter has exhibited high noise (radio beacons, reaction wheel momentum changes, *etc.*) and thus cannot be used quantitatively, only qualitatively. MinXSS-2 ground testing does not exhibit the same noise characteristics and should have reduced fast counter noise on-orbit.

As an alternative to estimating the true spectrum count rate on-orbit, one can use the merit function in Equation 8, *£*, where $n$ is the variable to estimate the spectrally integrated slow counter count rate without dead-time depression, in units of cps. The minimum of *£* with the restriction that ${C}_{s} < n < \frac{1}{{\tau}_{ds}}$ should yield the input count rate, ${n}_{\mathrm{min}}$, or “true” count rate that best estimates the measured spectrally integrated slow count rate, ${C}_{s}$. It is apparent from Figure 8 that the model function ${C}_{\mathrm{model}}$ is not monotonic, nor uniquely defined for input count rates in the interval $0 < n < \infty$. Thus, to obtain a feasible result, one must restrict the search domain to be between ${C}_{s} < n < \frac{1}{{\tau}_{ds}}$. For $n > \frac{1}{{\tau}_{ds}}$, other effects such as pile-up must also be corrected for. Additionally, the quantity of the difference between the observed count rate and the model count rate is squared to ensure positive concavity, and thus the realization that ${n}_{\mathrm{min}}$ will be the best fit result:

8 $$\begin{aligned} { \biggl[ \textit{\pounds} \biggl({C}_{s} < n < \frac{1}{{\tau }_{ds}}, {C}_{s}, { \tau}_{ds}\biggr) \biggr]}_{\mathrm{min}} =& { \biggl[ { \biggl[{C}_{s} - {C}_{\mathrm{model}}\biggl({C}_{s}< n < \frac{1}{{\tau}_{ds}}, {\tau}_{ds}\biggr) \biggr]}^{2} \biggr]}_{\mathrm{min}} \\ =& { \bigl[ { \bigl[{C}_{s} - n{e}^{-n{\tau}_{ds}} \bigr]}^{2} \bigr]}_{\mathrm{min}} . \end{aligned}$$

The ratio of the best-fit value to the actual spectrum summed count rate yields a correction factor $P$, where $P = \frac {{n}_{\mathrm{min}}}{{C}_{s}}$, which can be multiplied by each binned count rate in the spectrum to adjust for dead-time during the respective integration. Using this technique, the MinXSS data utility range can be extended to count rates determined by ${C}_{s} < \frac {1}{{\tau}_{ds}}$. As a proxy to indicate when these corrections will be needed, correlated XP data or the GOES/XRS flux levels can be used.

Early MinXSS-1 data from different GOES levels have been used to estimate the blue vertical axis in Figure 8, which roughly relates the GOES 0.1 – 0.8 nm ($\mbox{W}\,\mbox{m}^{-2}$) flux levels to the spectrally integrated MinXSS counts. The X123 counts are totaled across the entire operational spectral bins ($E_{\mathrm{ph}} \gtrsim 0.8~\mbox{keV}$) and not limited to the corresponding GOES 0.1 – 0.8 nm band (1.55 – 12.4 keV). This relation is to serve as a general guide of what count rates one would expect for specific GOES levels and can plan for X123 effects as necessary (photon peak pile-up, dead-time, *etc.*). The MinXSS-2 estimates are modeled counts based on the response functions and process described in Section 4.3, in Equation 2, where the photon source term, $S$, is generated from inverting the count spectrum measured with MinXSS-1. The rough estimates of the GOES levels corresponding to the MinXSS-2/X123 count rate are displayed as the red vertical axis in Figure 8. Examples of the count rate and estimated photon flux as a function of GOES levels from the early aspects of the MinXSS-1 mission are discussed in Section 5.

### MinXSS Data Products

The publicly available MinXSS data can be accessed on the MinXSS website http://lasp.colorado.edu/home/minxss/. These data will include Level 0c, Level 0d, and Level 1 – 5 products, and one of the most relevant products will be the Level 1 spectral irradiance (photons $\mbox{s}^{-1}\,\mbox{cm}^{-2}\,\mbox{keV}^{-1}$). Measured count rates, spacecraft position, Sun-Earth distance, pointing information, *etc.*, are also included. MinXSS data processing software to convert raw data into science-quality data will be incorporated into SSW soon.

## MinXSS-1 Solar Measurements from GOES A5–M5 Levels

The MinXSS/X123 spectrometer prototype was space-flight verified on two NASA sounding rocket flights for the calibration of the SDO/*Extreme Variability Experiment* (EVE: Woods *et al.*, 2012) and returned high-quality science data for two 5-minute periods (Caspi, Woods, and Warren, 2015). Thus, we have confidence in the MinXSS CubeSat versions of the X123 spectrometers to return high-quality data. This was reaffirmed with the first few months of data downlinked from the MinXSS-1 mission. Over the early phases of the mission, the X-ray flux has been as low as GOES ${\approx}\,\mbox{A5}$ and as high as GOES M5.0 during a flare. The times of the corresponding GOES levels analyzed in this article are

- A5 from 29 June 2016 10:29:32 – 01 July 2016 22:55:53 UT,
- B5 from 23 July 2016 01:15:05 – 01:39:45 UT,
- C2.7 from 08 July 2016 – 00:55:04 – 00:58:44 UT,
- M1.2 from 21 July 2016 – 01:50:01 – 01:53:31 UT,
- M5.0 from 23 July 2016 – 02:10:05 – 02:13:46 UT.

These data have been filtered, and only data that pass our “science quality” check (minimal background levels, particle events, non-South Atlantic Anomaly times, *etc.*) are analyzed below. These filter checks also isolate eclipse-time data with only thermal noise apparent in the spectrum, which result in ${\approx}\,2\,\mbox{--}\,5~\mbox{cps}$ across the entire spectrum. Other external (astronomical) soft-X-ray flux contributions to the MinXSS/X123 background are negligible because of the small instrument aperture. The solar flare times are centered on the flare peak total count rate in the MinXSS/X123 spectrum. These solar fluxes have all been corrected for dead-time losses using Equation 8 and result in MinXSS-1/X123 count rate levels of 26 – 9 100 cps. The dead-time losses were ${\approx}\,11\%$ for the M5.0 flare, ${\approx}\,3\%$ for the M1.2 flare, ${\approx}\,0.7\%$ for the C2.7 flare, and less than ${\approx}\,0.5\%$ for B GOES levels and lower. This demonstrates that the MinXSS spectrometer has the capability to cover a wide range of solar X-ray flux levels. XP responds identically to the increasing GOES flux, with background-subtracted signals in the range of 801 – 616 928 fC. This confirms the XP and X123 nominal operation.

Owing to the X123 noise sources mentioned off-diagonal elements of the X123 response, the lower end of the MinXSS-1/X123 spectra valid for scientific analysis is ${\approx}\,0.8~\mbox{keV}$. The sharply decreasing solar flux for high energies and the MinXSS small aperture result in an effective high-energy limit of ${\approx}\,12~\mbox{keV}$. Even for flares as large as M5.0, the flux is not large enough to produce a statistically significant count rate above 12 keV before effects such as detector dead-time and pile-up hinder the accuracy of the spectra. Thus, our MinXSS-1/X123 has an effective solar flux energy range of 0.8 – 12 keV and should return quality data up to low X-class flares if corrected for dead-time and pile-up effects. The MinXSS-2/X123 Fast SDD spectrometer with a nominal slow channel peaking time of 1.2 μs is four times faster than the nominal 4.8 μs peaking time, thus one would expect roughly four times the count rate before inaccurate spectra. But the MinXSS-1/X123 Be window is much thicker than the MinXSS-2/X123 window (24.5 *vs*. 11.2 μm), making comparisons nonlinear. Early estimates put the MinXSS-2/X123 maximum GOES level at X-class solar flares. Future data will reveal the full relation, keeping in mind that the axis on Figure 8 is a rough estimate and that the spectral distribution of photons is nonlinear *vs*. GOES flux levels.

**Figure 9:**
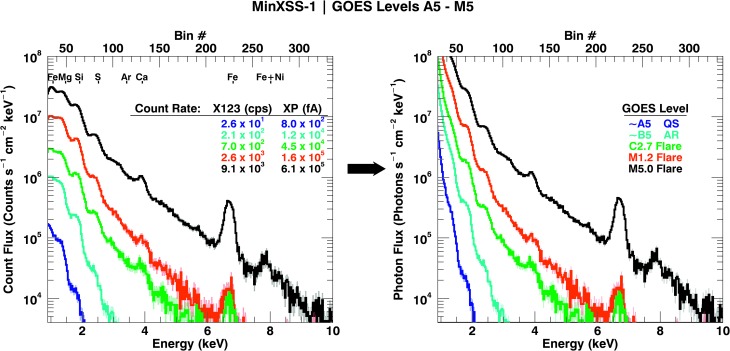
MinXSS-1 X123 solar measurements (*solid lines*) from GOES A5 to M5 levels (${\approx}\,5\times 10^{-8}\,\mbox{--}\,5\times 10^{-5}~\mbox{W}\,\mbox{m}^{-2}$). The *left plot* is the mean count flux, and the *right plot* is the estimated photon flux. The uncertainties are depicted as the *shaded region* around the measurements. This demonstrates the dynamic range of the MinXSS-1 spectrometer and the variation in spectral features for increasing solar flux levels. The “bumps” in the spectrum are due to groups of dominant emission lines from ionized Fe near 1.2 keV and 6.7 keV, Mg near 1.7 keV, Si around 2.1 keV, S by 2.7 keV, Ar (or lack thereof) near 3 keV, Ca by 4 keV, and the Fe+Ni complex at 8 keV. These features can be used as elemental abundance probes to assess deviations from the traditional “coronal” abundance values during various solar conditions.

The spectral photon flux estimates in Figure 9 demonstrate the drastic changes in the soft-X-ray spectra. The X123 soft X-ray spectrum measured changes by orders of magnitude over a few GOES level changes. Additionally, the XP fA signal (converted from the measured DN signal) scales with the GOES flux levels A5 to M5. This provides a consistency check for the X123 spectral signal. While the qualitative nature of this change is not new, the quantitative determination of the magnitude of this change is relevant. Table 2 lists the X123 and XP count rates as a function of GOES class and is plotted in Figure 9. The measured XP signal was compared to the X123 estimated XP signal by computing the estimated XP signal from the X123 estimated photon flux. The resulting “X123 modeled” XP signal is then compared to the measured XP signal. The XP measured and “X123 modeled” XP signal agree to within ${\approx}\,4\%$ except for the GOES A5 measurement. At low GOES levels, the XP signal becomes comparable to the thermal photodiode noise and leads to underestimated signals. This is apparent in the quiet-Sun (QS) emission measure inferences discussed in detail in Section 5.3.

**Table 2:** MinXSS-1 count rate and photon energy flux of observations from GOES A5–M5 levels. Ratio_B5_ is the count rate value of the corresponding row divided by the B5 count rate.

GOES level	X123_measured_ (counts s^−1^)	Ratio_B5_ X123	X123 total photon energy flux ${\geq}\,1~\mbox{keV}$ at 1 AU (W m^−2^)	XP_measured_ (fA)	Ratio_B5_ XP	XP_X123-predicted_ (fA)	$100\times(\Delta_{\mathrm{XP}}/\mbox{XP}_{\text{measured}})$
QS ≈ A5	2.63 × 10^1^	1.25 × 10^−1^	4.6 × 10^−6^	8.01 × 10^2^	6.67 × 10^−2^	1.33 × 10^3^	−66.35%
AR ≈ B5	2.10 × 10^2^	1.00 × 10^0^	4.6 × 10^−5^	1.20 × 10^4^	1.00 × 10^0^	1.15 × 10^4^	4.2%
C2.7 flare	7.01 × 10^2^	3.33 × 10^0^	1.5 × 10^−4^	4.53 × 10^4^	3.77 × 10^0^	4.36 × 10^4^	3.7%
M1.2 flare	2.60 × 10^3^	1.23 × 10^1^	5.5 × 10^−4^	1.65 × 10^5^	1.37 × 10^1^	1.61 × 10^5^	2.7%
M5.0 flare	9.1 × 10^3^	4.38 × 10^1^	2.1 × 10^−3^	6.18 × 10^5^	5.14 × 10^1^	6.16 × 10^5^	0.3%

A particularly interesting feature in the X123 spectrum are the two “bumps” near 1.7 keV that are due to Mg XI and Mg XII line-groups, and around 2.1 keV from Si XIII and Si XIV lines. These bumps persist for all GOES levels and provide useful diagnostics for element abundance estimations. The Fe XXIV and Fe XXV line complex near 6.7 keV is prominent for the GOES flares of levels C2.7 and higher. This line complex is well suited for estimating the Fe abundance modification during solar flares *vs*. the QS (non-large-flaring Sun). Only for the brightest flares (M5.0 and M1.2 in this article) is the Fe–Ni complex near 8.0 keV pronounced and suitable for analysis. For smaller flares, the signal is not statistically significant to infer physical properties from this feature. These solar measurements demonstrate the dynamic range of the MinXSS instruments.

### Soft X-Ray Energy Content and Correlation to GOES/XRS

**Figure 10:**
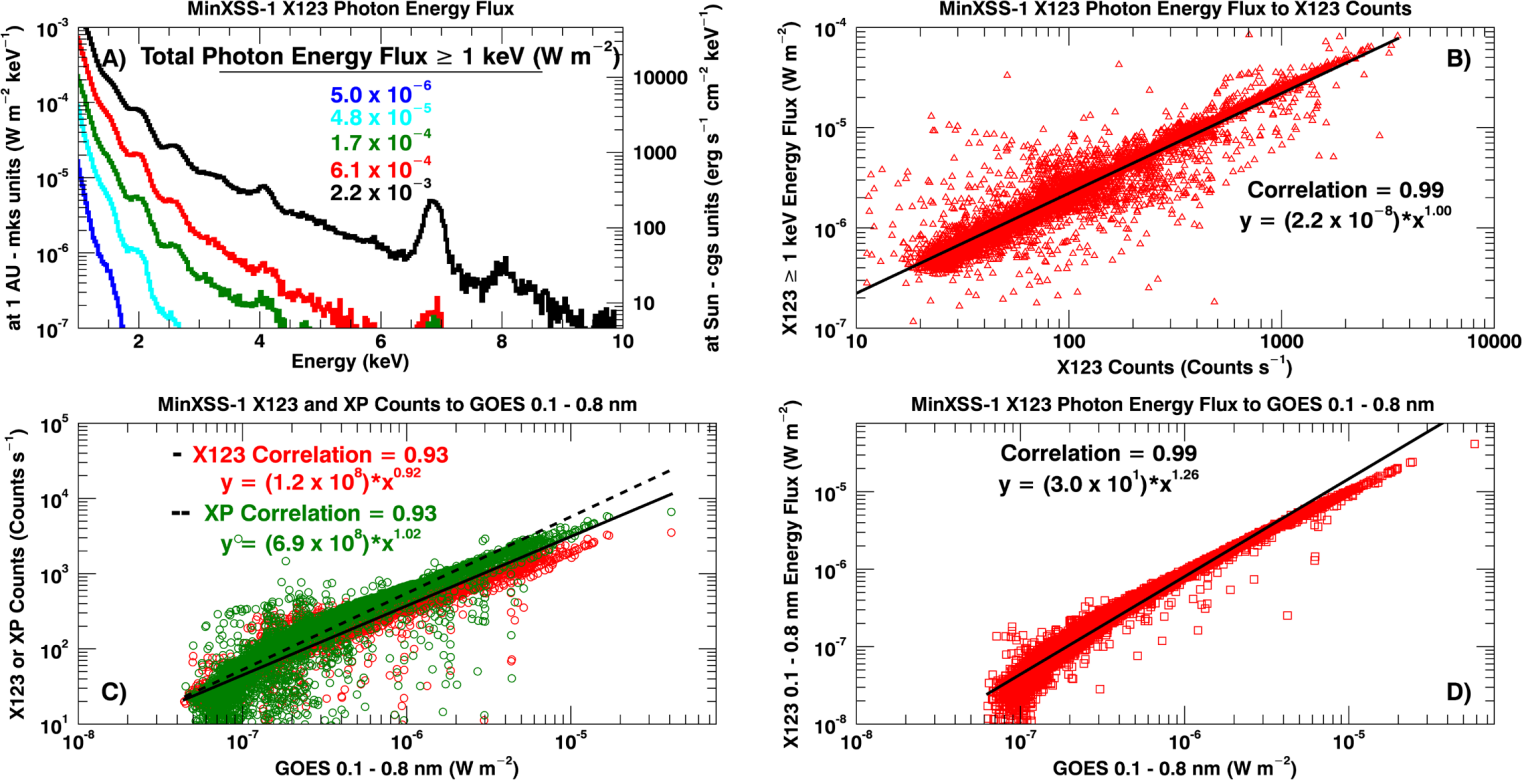
(**A**): MinXSS-1/X123 derived photon energy flux at 1 AU (in MKS units – $\mbox{W}\,\mbox{m}^{-2}\,\mbox{keV}^{-1}$) and scaled back to the solar surface (in CGS units – $\mbox{erg}\,\mbox{s}^{-1}\,\mbox{cm}^{-2}\,\mbox{keV}^{-1}$). The total energy flux at 1 AU as measured by MinXSS-1 for photon energies ${\geq}\,1~\mbox{keV}$ is listed for the GOES ${\approx}\,\mbox{A5}$ (*blue*), B5 (*cyan*), C2.7 (*green*), M1.2 (*red*), and M5.0 (*black*) class observations. (**B**) – (**D**): Scatter plots, correlation coefficients, and linear fit of MinXSS-1/X123 photon energy ${\geq}\,1~\mbox{keV}$ to count rate (panel **B**), MinXSS-1/X123/XP count rate to GOES 0.1 – 0.8 nm flux (panel **C**) and MinXSS-1/X123 photon energy flux integrated from 0.1 – 0.8 nm (${\approx}\,1.55\,\mbox{--}\,12.4~\mbox{keV}$) to GOES/XRS 0.1 – 0.8 nm flux (panel **D**) all show very strong correlations, indicating only small systematic differences between the two datasets.

A main benefit of conducting routine spectrally resolved soft X-ray measurements is the ability to calculate and track the spectral distribution of the energy flux. This information is important for models of the Earth’s atmospheric response to solar radiative forcing, solar flare analyses, and comparison of the solar X-ray flux to other stars. Table 2 lists the integrated soft X-ray energy flux (multiplying the energy flux by the X123 bin width and summing over photon energy bins) above 1 keV estimated at 1 AU from MinXSS-1/X123 measurements for the main five GOES levels. This information is also presented in Figure 10A. Panel B of Figure 10 displays the fitted power-law relationship, $y = a(x^{b})$, between the MinXSS-1/X123 total counts and the total radiative energy greater than 1 keV, which is not quite linear. The data in the scatter plots (Figure 10B – D) are from 10 June – 30 November 2016. The coefficient of $2.2 \times 10^{-8}~\mbox{J}\,\mbox{counts}^{-1}\,\mbox{m}^{-2}$ can be used to obtain a rough estimate of the energy content from the X123 total counts alone. A large portion of this energy (up to an order of magnitude) resides below 2 keV, and this is apparent when comparing to the corrected (divided by 0.7) GOES/XRS 0.1 – 0.8 nm flux, see Figure 10A.

There is a positive correlation between the 0.1 – 0.8 nm (${\approx}\,1.55\,\mbox{--}\,12.4~\mbox{keV}$) energy flux calculated from MinXSS-1/X123 and GOES/XRS (Figure 10D). The XP count rate ($\mbox{DN}\,\mbox{s}^{-1}$) has a nearly linear relationship to the GOES/XRS 0.1 – 0.8 nm flux, especially above GOES B1 levels, and is useful as a proxy to the GOES/XRS measurements. This is expected as XP tracks the total soft X-ray energy incident on the MinXSS-1 aperture (the number of electron-hole pairs generated is proportional to $E_{\mathrm{ph}}$). Exact linearity is not expected between X123 count rate and GOES/XRS energy flux because X123 is photon counting. These results validate the dynamic response of the MinXSS-1/X123 and XP to the solar flux. The next section discusses the feasibility of extracting physical information from model fits of the MinXSS/X123 data to estimate plasma temperature, density, emission measure, and elemental abundances.

### Spectral Parametric Fits

**Figure 11:**
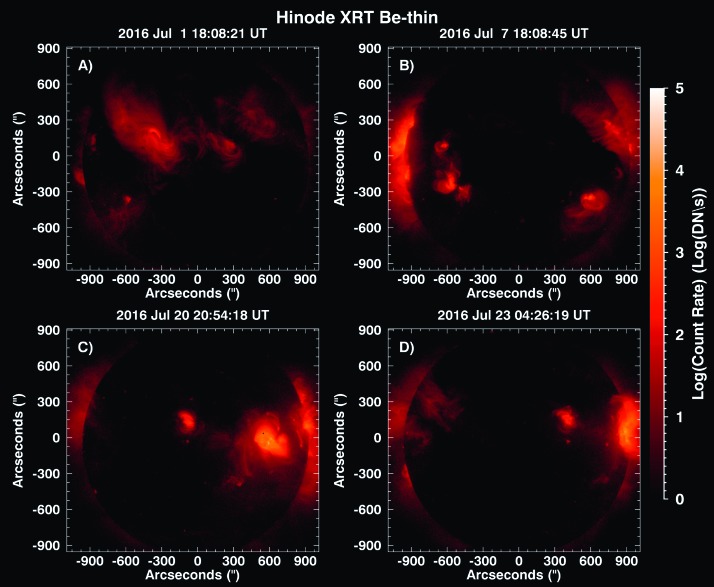
(**A**) Log-scaled count rate *Hinode*/XRT Be-thin full-Sun images near the time of the MinXSS-1 observed QS. (**B**), (**C**), and (**D**) Pre-flare times for the C2.7, M1.2 and M5.0 flares, respectively. The XRT images provide information on the spatial distribution of the soft X-ray emitting plasma, since MinXSS measurements are integrated across the entire FOV.

*Hinode*/XRT Be-thin images are displayed in Figure 11 to provide qualitative spatial context for the X-ray emission that MinXSS detects for non-large-flaring times. The XRT Be-thin serves as the closest synoptic filter analog to the MinXSS spectral response. The four panels in Figure 11 are the closest XRT full-Sun synoptic images in time to the QS (Figure 11A), pre-flare C2.7 (Figure 11B), pre-flare M1.2 (Figure 11C), and pre-flare M5.0 (Figure 11D) times. It is clear that when active regions are present, their emissions dominates the MinXSS count rates (this is due to the hotter plasma content). We are currently working to cross-calibrate the two instruments to develop quantitative relationships. With this knowledge, we proceed to demonstrate an example of how MinXSS data can be used to extract physical information on the X-ray emitting regions.

The unique spectrally resolved measurements of MinXSS are suitable for parametric fits of single-temperature (1T), two-temperature (2T), multi-temperature (multi-T), and differential emission measure (DEM) models to estimate the plasma conditions. QS, AR, and flare data over the possible total six-year MinXSS mission combined with data from the observatories mentioned in Section 1.1 can be used to address current questions in solar physics. We have performed a series of parametric fits using the Objective Spectral Executive (OSPEX) (https://hesperia.gsfc.nasa.gov/ssw/packages/spex/doc/ospex_explanation.htm) programming suite in Solar Software, which utilizes the Chianti atomic database (Del Zanna *et al.*, 2015; Young *et al.*, 2016), on the seven sets (includes pre-flare times) of data described earlier from GOES A5 to M5 levels. The uncertainties calculated on the fit parameters in this article are basic OSPEX-returned fit uncertainties, based on the curvature matrix, which assumes that the curvature has a local Gaussian shape.

**Figure 12:**
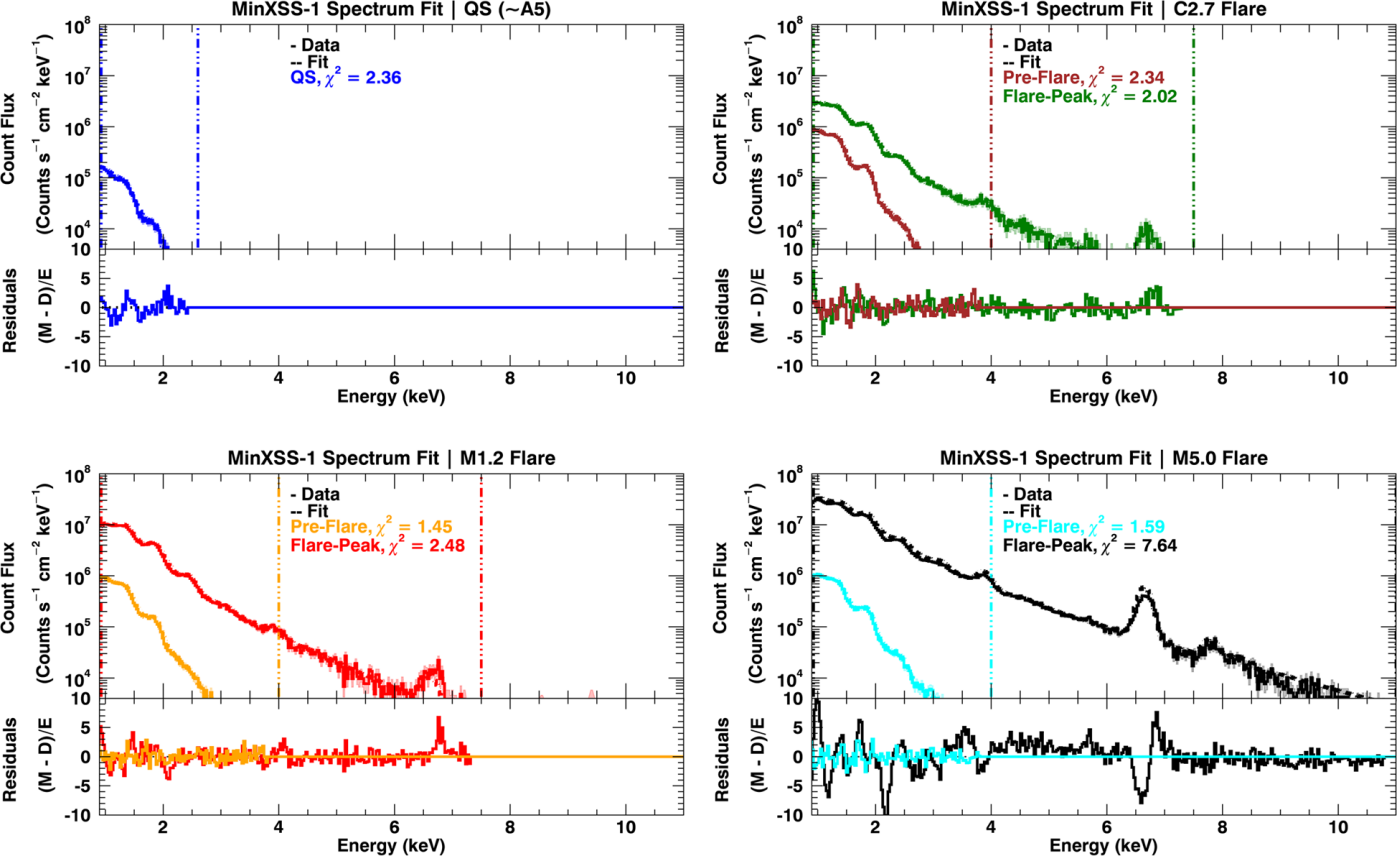
MinXSS-1 X123 count flux solar measurements (*solid lines*) with the best-fit spectra overlaid (*dashed lines*) for temperature and emission measures derived using the OSPEX suite. The residuals are listed also ($M = \mbox{model}$, $D = \mbox{data}$, and $E = \mbox{uncertainty}$). The *shaded regions* indicate the uncertainties in the count flux. A 2T model with a selected elemental abundance fit separately (2T-AllFree). The best-fit parameters with their uncertainties are listed in Table 4 and Table 5. A 2T model is used for non-large-flaring times (QS and pre-flare), and an additional 2T model is added to compensate for the radiative enhancement during the flare-peak times. The *vertical dash-triple-dotted lines* show the high- and low-energy limits for the spectral fits.

**Figure 13:**
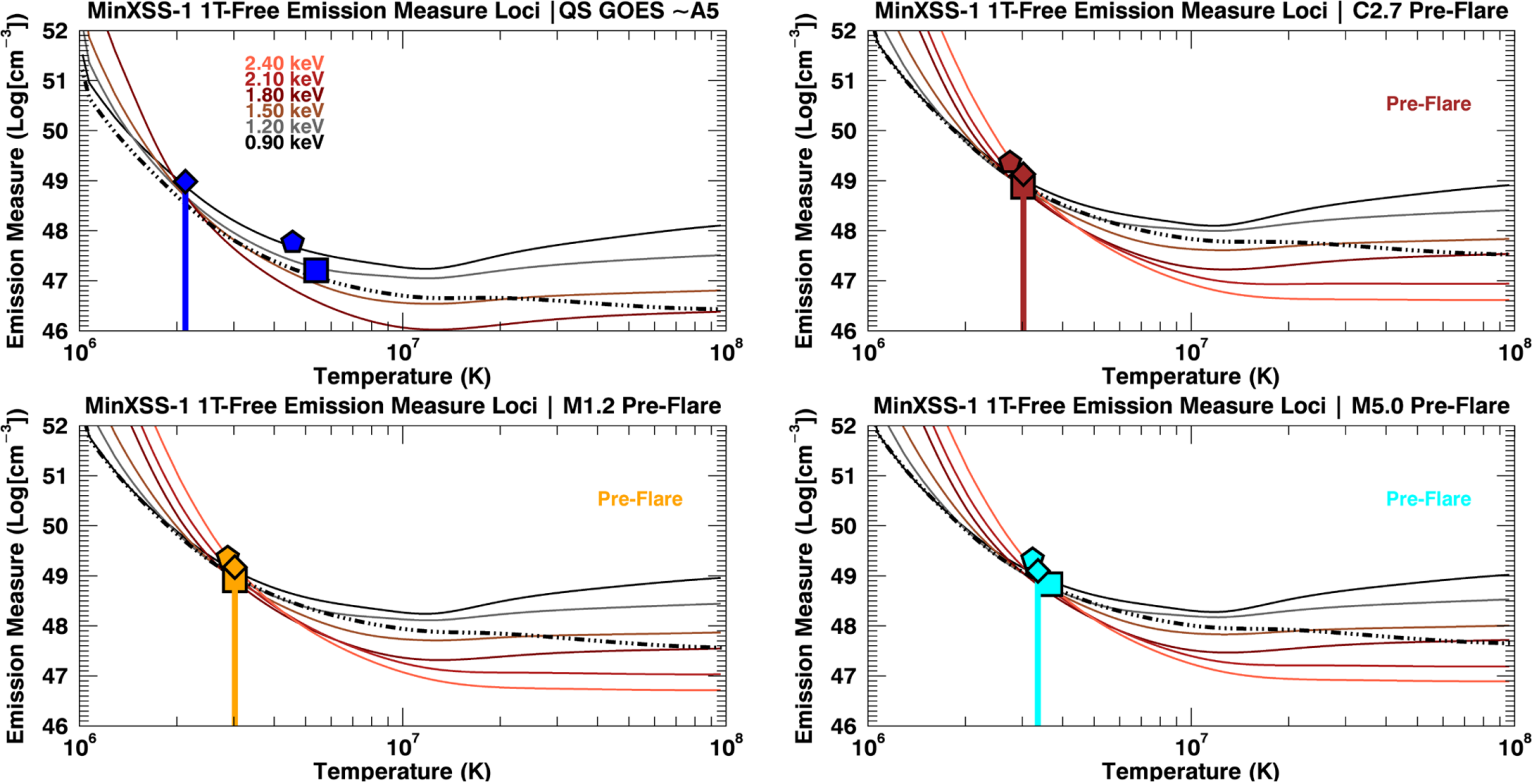
Volume emission measure loci (Em loci) plots with MinXSS-1 OSPEX 1T-Free fit parameters overplotted as delta functions in temperature, with filled diamonds indicating the emission measure value for the non-large-flaring Sun (pre-flare). These MinXSS-1 Em loci and fit parameters correspond to the spatial distribution captured by *Hinode*/XRT in Figure 11. The *solid colored lines* correspond to X123 counts summed to 0.3 keV wide energy bins, and the *dash-triple-dotted lines* represent the XP loci. The rainbow keV values in the top left plot indicate the color code for the minimum energy bin used for each X123 Em loci. The Em loci indicate the maximum emission if all the plasma were isothermal for each of the summed energy bins. GOES-averaged values are listed for photospheric (*pentagon*) and coronal (*square*) abundances. The plot of X123 1T-Free fits are to demonstrate: 1) agreement with the overlapping X123 Em loci, 2) agreement with the overlapping XP Em loci, and 3) consistency with the GOES/XRS isothermal estimation except for low GOES levels like the ${\approx}\,\mbox{A5}$ levels (this is due to the nonlinearity of GOES for low flux levels).

**Figure 14:**
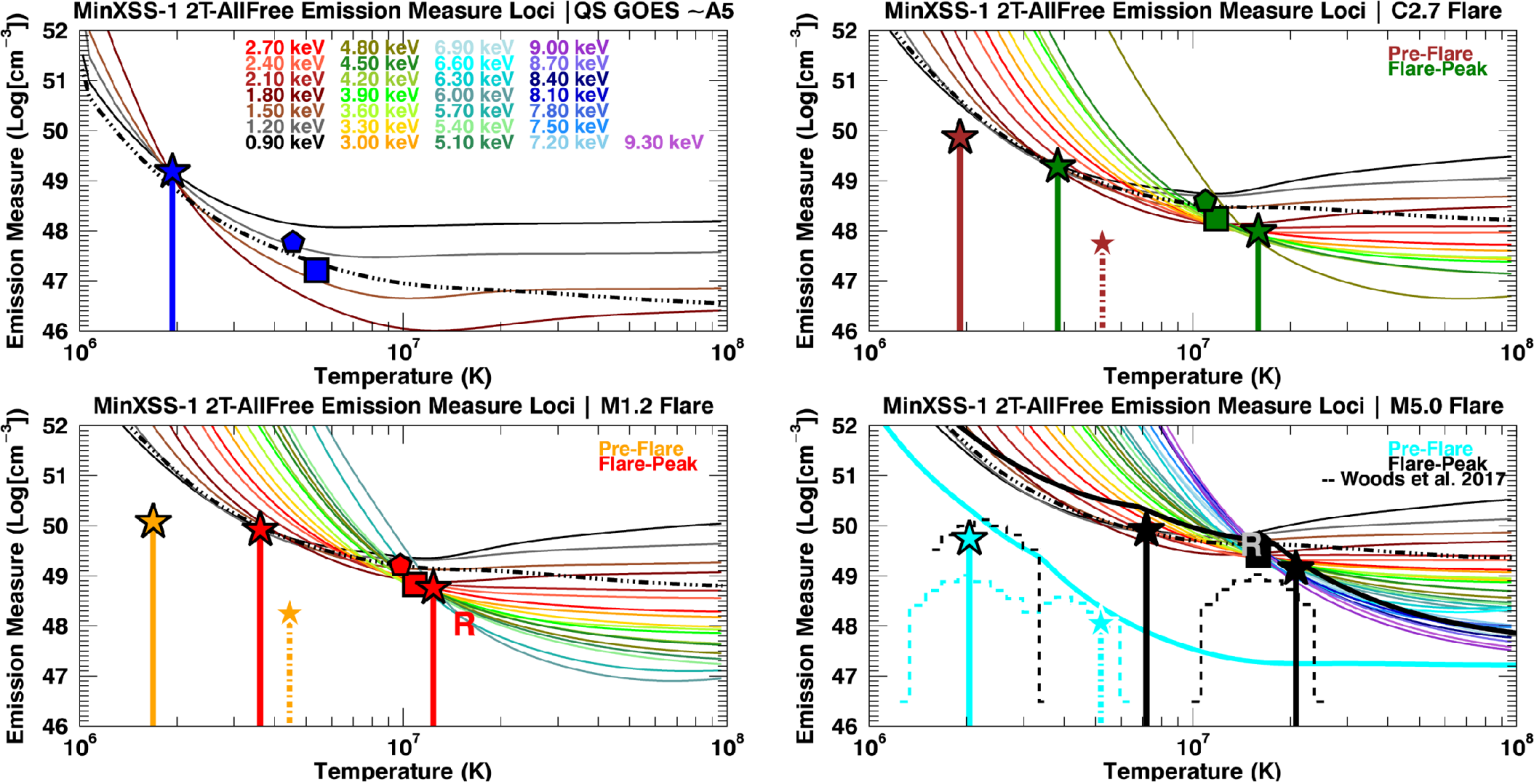
Em loci plots with MinXSS-1 OSPEX 2T-AllFree fit flare-peak and QS parameters overplotted as delta functions in temperature, with filled stars indicating the emission measure value. The solid line delta functions are well constrained by the MinXSS data. The pre-flare *dash-dotted* delta function without the black outline indicates that the hotter-dimmer component is less well constrained by MinXSS data. The *thin solid colored lines* correspond to X123 counts summed to 0.3 keV wide energy bins, and the *dash-triple-dotted lines* are the XP EM loci. The rainbow keV values in the top left plot indicate the color code for the minimum energy bin use for each X123 Em loci. The X123 and XP Em loci are consistent. The M5.0 flare fit results from Woods *et al.* (2017) are overlaid as the *dashed histograms* in the bottom right panel. The thick black Em loci show the M5.0 flare and the thick cyan Em loci the pre-flare; both are the minimum of all the individual energy bins corresponding to the spectral model used in the Woods *et al.* (2017) analysis. GOES averaged values are listed for photospheric (*pentagon*) and coronal (*square*) abundances. RHESSI values for the M1.2 and M5.0 flare are indicated by the $R$.

We have used four models to fit the respective observations for comparisons between models. The best-fit values are listed in Table 3, Table 4, Table 5, Figure 12, Figure 13, and Figure 14. In this analysis we denote a FIP-bias value of 1 equal to photospheric, which would have values similar to those in Caffau *et al.* (2011) and a FIP-bias of 4 equal to the traditional “coronal” abundance, which are those of Feldman (1992). The first model is a simple one-temperature, fixed coronal abundance (1T-Coronal, $A_{\mathrm{FIP}} = 4$) model, which did not fit the data suitably for any of the seven data sets. There were large discrepancies for the elemental features labeled in Figure 9. The second model is a single-temperature model in which the abundance of low-FIP elements is allowed to vary through a single multiplicative factor (FIP-bias). This model is called 1T-Free, but it underestimates the pre-flare flux for photon energies greater than ${\approx}\,2.5~\mbox{keV}$. The same 1T-Free model did not fit the flare components well either. For the times without substantial counts for X123 energy bins ${>}\,2.5~\mbox{keV}$, such as the QS flux (GOES ${\approx}\,\mbox{A5}$), the 1T-Free model provides an adequate fit.

**Table 3:** MinXSS-1 2T-Free (one FIP-bias scale factor) spectral fits of observations from GOES A5–M5 levels. The uncertainties in the fit parameters are listed in parenthesis. Two asterisks highlight that the dimmer and hotter second component inferred from pre-flare data is near the limit of the MinXSS plasma diagnostic capabilities and thus not constrained as well.

GOES level	Observation times	EM_1_ (${10}^{49}~\mbox{cm}^{-3}$)	T_1_ (MK)	EM_2_ (${10}^{49}~\mbox{cm}^{-3}$)	T_2_ (MK)	FIP-bias_12_ (1 = photospheric) (4 = coronal)
QS ≈ A5	2016-Jun-29 10:29:32 – 2016-Jul-01 22:55:53	19.9 (9.2)	1.17 (0.08)	0.21 (0.07)	2.43 (0.12)	3.48 (0.46)
C2.7 pre-flare (≈ B3)	2016-Jul-07 23:41:03 – 2016-Jul-08 00:04:24	10.8 (0.75)	1.70 (0.02)	*0.12 (0.01)*	*4.58 (0.08)*	2.89 (0.10)
C2.7 flare	2016-Jul-08 00:55:04 – 2016-Jul-08 00:58:44	1.21 (0.04)	4.28 (0.07)	0.10 (0.005)	14.91 (0.27)	1.41 (0.06)
M1.2 pre-flare (≈ B3)	2016-Jul-20 18:04:13 – 2016-Jul-20 18:28:53	8.73 (0.81)	1.82 (0.04)	*0.13 (0.02)*	*4.68 (0.14)*	2.17 (0.09)
M1.2 flare	2016-Jul-21 01:50:01 – 2016-Jul-21 01:53:31	6.21 (0.11)	4.05 (0.03)	0.46 (0.01)	12.97 (0.12)	1.41 (0.03)
M5.0 pre-flare (≈ B5)	2016-Jul-23 01:15:05 – 2016-Jul-23 01:39:45	7.68 (0.80)	1.86 (0.05)	*0.18 (0.02)*	*4.80 (0.12)*	2.21 (0.09)
M5.0 flare	2016-Jul-23 02:10:05 – 2016-Jul-23 02:13:46	17.10 (0.13)	4.86 (0.02)	2.22 (0.02)	19.67 (0.08)	0.98 (0.01)

**Table 4:** Temperature and emission measure values from MinXSS-1 2T-AllFree (separate elemental abundance scale factors) spectral fits of observations from GOES A5–M5 levels that are plotted in Figure 12. The best-fit abundances are listed in Table 5. The uncertainties in the fit parameters are given in parenthesis. Two asterisks highlight that the dimmer and hotter second component inferred from pre-flare data is near the limit of the MinXSS plasma diagnostic capabilities and thus not constrained as well.

GOES level	EM_1_ (${10}^{49}~\mbox{cm}^{-3}$)	T_1_ (MK)	EM_2_ (${10}^{49}~\mbox{cm}^{-3}$)	T_2_ (MK)
QS ≈ A5	15.2 (0.21)	1.93 (0.05)	–	–
C2.7 pre-flare (≈ B3)	7.29 (0.59)	1.91 (0.04)	*0.05 (0.01)*	*5.25 (0.23)*
C2.7 flare	1.91 (0.12)	3.82 (0.08)	0.09 (0.006)	15.92 (0.45)
M1.2 pre-flare (≈ B3)	11.90 (2.5)	1.69 (0.08)	*0.18 (0.03)*	*4.45 (0.16)*
M1.2 flare	8.27 (0.41)	3.61 (0.05)	0.57 (0.02)	12.38 (0.16)
M5.0 pre-flare (≈ B5)	5.44 (0.78)	2.04 (0.08)	*0.11 (0.03)*	*5.19 (0.26)*
M5.0 flare	8.16 (0.11)	7.18 (0.02)	1.38 (0.02)	20.78 (0.16)

**Table 5:** Separate abundance values are in abundance ratio units of coronal/photospheric, where the coronal values are taken from Feldman (1992) and the photospheric values are adopted from Caffau *et al.* (2011) from MinXSS-1 2T-AllFree spectral fits of observations from GOES A5–M5 levels that are plotted in Figure 12. Elemental abundances that were fixed during fitting have the word “fixed” in parenthesis in place of an uncertainty. These values were fixed during fitting when there were insufficient counts in the corresponding spectral feature to ascertain an abundance. The abundances of He, C, O, F, Ne, Na, Al, and K were fixed at photospheric values. The best-fit temperatures and emission measures are listed in Table 4.

Element FIP (eV)	Fe 7.90	Ca 6.11	S 10.36	Mg 7.65	Si 8.15	Ar 15.76	Ni^a^ 7.64
QS ≈ A5	0.06 (1.75)	4.00 (fixed)	1.29 (fixed)	1.43 (0.18)	3.90 (0.89)	1.20 (fixed)	0.06 (1.88)
C2.7 pre-flare (≈ B3)	2.26 (0.28)	4.00 (fixed)	1.32 (0.64)	2.60 (0.10)	3.39 (0.20)	1.20 (fixed)	2.43 (0.30)
C2.7 flare	0.78 (0.08)	3.18 (0.47)	1.11 (0.69)	1.06 (0.07)	1.38 (0.07)	0.81 (0.28)	0.84 (0.08)
M1.2 pre-flare (≈ B3)	1.94 (0.23)	4.00 (fixed)	0.98 (0.74)	2.30 (0.13)	1.80 (0.14)	1.20 (fixed)	2.08 (0.25)
M1.2 flare	1.00 (0.06)	1.64 (0.26)	1.05 (0.67)	1.59 (0.06)	1.34 (0.04)	0.75 (0.16)	1.08 (0.06)
M5.0 pre-flare (≈ B5)	2.39 (0.28)	4.00 (fixed)	1.37 (0.64)	2.26 (0.12)	2.41 (0.17)	1.20 (fixed)	2.56 (0.29)
M5.0 flare	1.85 (0.02)	4.23 (0.11)	0.90 (0.65)	1.62 (0.03)	0.80 (0.01)	1.10 (0.01)	1.98 (0.02)

^a^The Ni abundance was linked to the Fe abundance, therefore this is not a truly independent estimate of the Ni abundance.

The third model is a two-temperature component with a single multiplicative factor for the FIP-bias (2T-Free). These fits consistently produce satisfactory results with reduced $\chi^{2}$ between 1 – 4, except for the M5.0 flare. All pre-flare spectra in this article are fit over the times listed in Table 3 with a 2T-Free model. The flare-peak fits include an additional 2T-Free model that is fit to account for the additional radiation on top of the fixed model pre-flare values. In this method, the fixed pre-flare model during the peak-flare times serves as a background estimate. Thus, the result is a separate FIP-bias for the 2T-Free pre-flare and the flare-peak functions. Values near 4 for this fit class resemble traditional “coronal” values, and values near 1 are photospheric.

The abundance models are sensitive to the “bumps” from emission line-groups of Fe, Mg, Si, S, Ar, Ca, and Ni (where applicable) around ${\approx}\,1.2$, 6.7 and 8.1, 1.7, 2.1, 2.7, 3.0, 4.0, and 8.1 keV, respectively. For the 2T-Free model, a FIP-bias value of 3.48 is found for the QS spectra, for the pre-flare times, values between 2.0 – 3.5 are obtained, and lower values between ${\approx}\,1\,\mbox{--}\,1.41$ are obtained for the peak-flare spectral fits. It is clear that there is a difference in the estimated abundance from pre-flare to flare-peak, and the lower abundance during the flare is consistent with recent literature (Schmelz *et al.*, 2012; Dennis *et al.*, 2015; Woods *et al.*, 2017). The availability of the Fe and Fe+Ni complexes at 6.7 and 8.1 keV, respectively, provides clear diagnostics for the abundance factors, and they in turn are weighted more heavily for the flare abundances. The lower abundance is also in line with the theory of plasma from the lower atmosphere flowing up to the higher layers of the atmosphere and radiating in X-rays and UV.

The fourth model type is a two-temperature component where each of the elements, Fe, Ca, S, Mg, Si, Ar, and Ni, is allowed to vary (2T-AllFree) as long as there are sufficient counts in the X123 energy bins for the respective element line-group features in the spectrum. It is important to note that the Ni abundance scale factor is coupled to the Fe abundance scale factor. Thus, the Ni abundance is not a truly independent inference. This is heritage in the fitting routine for the Fe+Ni complex near 8.0 keV and does not strongly skew the fits. The 2T-AllFree temperature and emission measure results are listed in Table 4, the elemental abundance results are collected in Table 5, the spectral fits are presented in Figure 12 and the comparison to volume emission measure loci (Em loci) is made in Figure 14. The separate abundance values are given in abundance ratio units of coronal/photospheric, where the coronal values are taken from Feldman (1992) and the photospheric values are adopted from Caffau *et al.* (2011).

The QS spectra do not have enough counts for energies ${\geqq}\,2.5~\mbox{keV}$ and the features are not strong enough for a consistent fit, and thus the individual element abundance parameters are poorly constrained. For the pre-flare and flare-peak spectra, however, statistically significant abundance values can be deduced. Again the trend for a decrease in the abundance for the majority of the low-FIP elements (Fe, Si, Mg, Ni) during the flare-peak *vs*. the pre-flare fits is clear. Additionally, the observed individual element abundance variation provides further evidence for a more complicated fractionation process than a simple single FIP-bias scaling for all the low-FIP elements. Similar conclusions that abundances are more complex than a simple single FIP-bias scaling have been expressed in recent studies, such as in Schmelz *et al.* (2012) and Dennis *et al.* (2015).

This complexity is further complicated by the postulated decrease in abundance from the nominal coronal to photospheric ratio of Ar for the three flares studied in this article and Ca for two of the three flares. The abundances of Ar and Ca have been of recent interest in a *Hinode*/EIS spectrum (Doschek, Warren, and Feldman, 2015; Doschek and Warren, 2016), and MinXSS can provide an additional diagnostic in investigating any anomalous behavior. A more rigorous analysis of elemental abundance variations, solar flares, quiescent conditions, and active region evolution comprising DEM fits will be made in the future.

The MinXSS-1/X123 spectral fits of the non-flaring Sun are consistent with a dominant emission component between 1 – 3 MK with volume emission measure values near $10^{49}\,\mbox{--}\,10^{50}~\mbox{cm}^{-3}$, which is not surprising. To highlight the limit of inference in these simple 2T models, we include the more uncertain hotter (${\gtrapprox}\,4.5~\mbox{MK}$), dimmer (${\lessapprox}\,10^{48}~\mbox{cm}^{-3}$) secondary component. With this secondary component, the model fits the data well with reasonable $\chi^{2}$ values. Without this secondary temperature component, the 1T models underestimate the measured count rate above 2.5 keV, although a DEM fit could reconcile this excess. The ability of MinXSS data alone to constrain this contribution is limited (by the small effective area) and can only provide upper limits to the emission measure. We caution against the magnitude of the emission measure of this hot-dim component derived from 2T fits from MinXSS data alone and signify this with two asterisks in Table 3 and Table 4 on the fit parameters and a dash-dot delta function without a black outline in Figure 14. Conclusive evidence of its existence can be provided by simultaneous observations from other more sensitive instruments. The purpose of this article is to highlight the MinXSS capabilities, but this also encompasses uncovering the limitations.

We checked the estimated GOES/XRS 0.05 – 0.4 nm flux for the MinXSS 2T inferred pre-flare secondary hot-dimmer components using the goes_fluxes.pro Interactive Data language (IDL) code. The measured fluxes from the GOES 0.05 – 0.4 nm channel would have to be between at least 2.2, 2.9, and $4.2\times 10^{-9}~\mbox{W}\,\mbox{m}^{-2}$ (for photospheric abundances) for the pre-flare times of C2.7, M1.2, and M5.0 for this component to have a similar emission measure as the MinXSS 2T-AllFree fits. All measured GOES 0.05 – 0.4 nm fluxes were at about a factor of two below the estimated values. Spectral fits to accommodate the measured count rate above 2.5 keV are most likely reconciled by DEM fits of MinXSS data coupled with other soft X-ray data. This will be done soon.

AR hot components ($T \geqq 5~\mbox{MK}$) inferred from soft X-ray data have frequently been discussed (see Caspi, Woods, and Warren, 2015; Miceli *et al.*, 2012; Reale *et al.*, 2009; Schmelz *et al.*, 2009a,b, 2015, to state a few articles, and references therein). All of these studies have their respective limitations. Future measurements from the *Marshall Grazing Incidence X-ray Spectrometer* (MaGIXS) (Kobayashi *et al.*, 2011) sounding rocket and continued measurements from the *Focusing Optics X-ray Solar Imager* (FOXSI: Ishikawa *et al.*, 2014) sounding rockets as well as the NuSTAR satellite (Hannah *et al.*, 2016) will provide additional data to validate or refute the previous claims of the hot-dim plasma, or at the very least, will provide firm upper limits on its emission measure. The only substantial inkling seems to come from the *Extreme Ultraviolet Normal Incidence Spectrograph* (EUNIS-13: Brosius, Daw, and Rabin, 2014) and most recently from FOXSI (Ishikawa *et al.*, 2017) rocket flights. MinXSS data combined with the other soft X-ray and UV observatories can help to further constrain the existence of hot-dim plasma and investigate the solar plasma temperature distribution.

The peak-flare emission in this article is best described by a persistent cooler component between 3 – 7 MK plus a hotter contribution (${\geqq}\,13~\mbox{MK}$) that dominates at the higher energy flux. All flare data analyzed in this article cover about a time frame of 3 – 4 min centered on the peak soft X-ray emission times. Refinement of the MinXSS-1 spectral responses is a continuous endeavor, particularly for high-flux times, such as the M5.0 flare (which is currently the highest flux observation that we have analyzed thoroughly). The deviations near values of 5 between the uncertainty-normalized difference between the model and observations for the Si 2.1 and the Fe 6.7 keV features need further improvement.

A further check of the MinXSS-1 flare measurements is provided with a comparison to nearly simultaneous RHESSI measurements. RHESSI 1T fit emission measure and temperature results are overlaid as $R$ in Figure 14 for the M1.2 and M5.0 flares, which were the only flares in this article that were simultaneously observed by both MinXSS-1 and RHESSI. The RHESSI fits were performed in OSPEX, composed of a 1T thermal and a nonthermal (thick2) bremsstrahlung component. The spectra were fit from ${\approx}\,6~\mbox{keV}$ to the maximum photon energy with signal above the background (${\approx}\,30~\mbox{keV}$). The RHESSI thermal fit components yield values near the GOES estimates (White, Thomas, and Schwartz, 2005) and the MinXSS-1 Em loci curves (discussed in Section 5.3). RHESSI estimates a temperature of 15 MK for both flares and emission measure values of 0.1 and $2.5 \times10^{49}~\mbox{cm}^{-3}$, respectively, for the M1.2 and M5.0 flare. RHESSI fits indicate an elemental abundance FIP-bias factor of 2.1 and 1.3 for the M1.2 and M5.0 flares, respectively, which are both below traditional “coronal” values.

**Figure 15:**
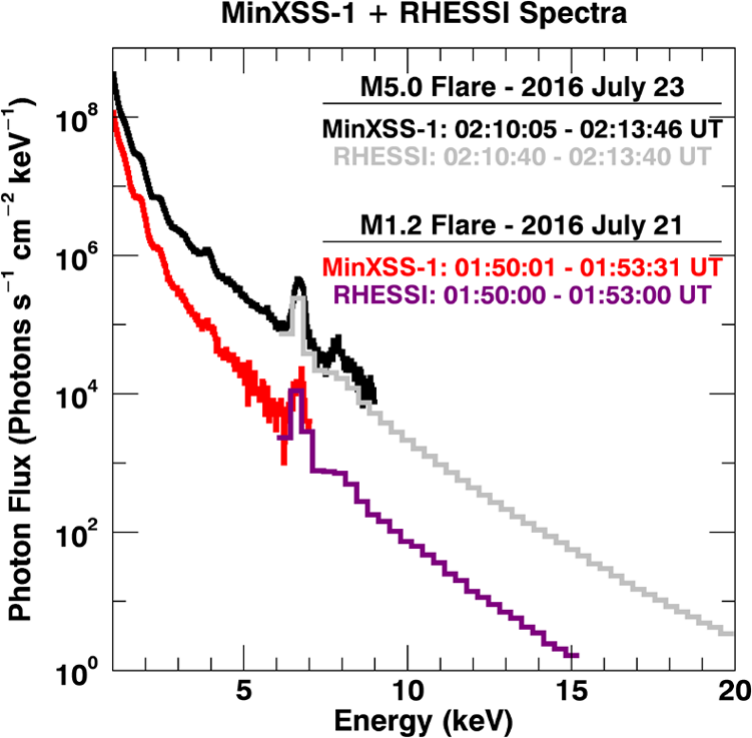
MinXSS-1 M1.2 and M5.0 flare photon flux spectra with RHESSI spectra overlaid. These nearly simultaneous measurements provide complete spectral coverage from 1 keV to the minimum detected flux from RHESSI and span eight orders of magnitude in flux. The main overlap between instruments for flares is near the 6.7 keV Fe complex. This comparison helps validate the MinXSS observations.

**Figure 16:**
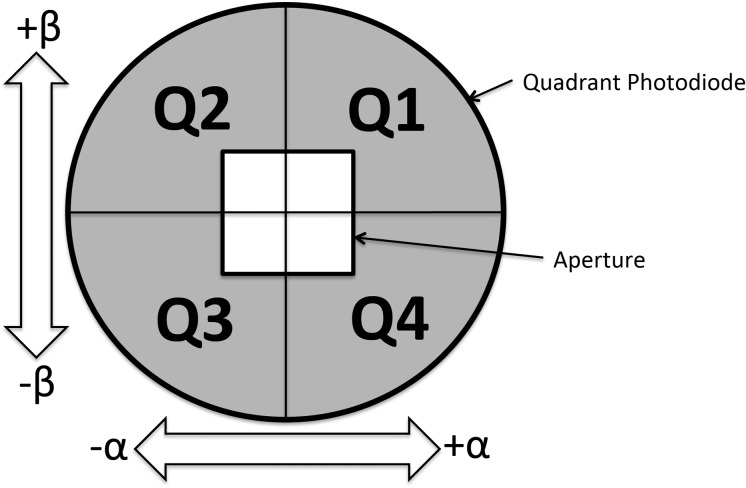
SPS quad diode layout demonstrating the orientation of the $\alpha$ and $\beta$ angles. The SPS square aperture is $5~\mbox{mm} \times 5~\mbox{mm}$.

It is not expected for MinXSS-1 and RHESSI to return the exact same temperature and emission measure values because the spectral responses, spectral resolution, effective areas, and consequential temperature sensitivities are all different, but one expects consistencies in RHESSI plasma parameters with MinXSS Em loci and the inferred photon flux. The MinXSS-1 and RHESSI photon flux in Figure 15 provides one of the few spectrally complete flare measurements from 1 keV to at least 15 keV. The combined MinXSS-1 and RHESSI dynamic range spans nearly 8 dex of photon flux, and there is an overlap near the 6.7 keV Fe complex. The ${\approx}\,8~\mbox{keV}$ Ni feature is also apparent in the MinXSS-1 spectra. Similarly, RHESSI data can be used to extract nonthermal contributions to the MinXSS-1 spectra for large flares. The discussion of the instrument complexities and a cross calibration between MinXSS and RHESSI is reserved for a future article (Amir *et al.*, in preparation).

### Emission Measure Loci

Emission measure loci (Em loci) provide a powerful diagnostic and a useful visualization tool to understand a spectral instrument capability to infer the plasma temperature distribution from a specific set of observations (Warren and Brooks, 2009; Landi and Klimchuk, 2010). Em loci can be calculated by taking the measured data (summed counts over a specified energy range for X123 or the total signal for XP) and dividing by the instrument temperature response calculated for that energy range, for the corresponding best-fit elemental abundance and emission model, $\mbox{Em loci} = \frac{\text{Counts}}{F(T)}$, where $F(T)$ is the temperature response and Counts is the signal in units corresponding to $F(T)$. Figure 14 displays the MinXSS-1/X123 Em loci for the QS and three flare times, along with the best-fit 2T-Free emission measure results. The different colors show the Em loci of the various summed 0.3 keV wide energy bins (counts from ${\approx}\,10$ X123 bins summed) with a significant count rate (${\geqq}\,0.05$ counts *per* second *per* nominal bin), which depict the energy ranges that best restrict the emission at specific temperatures. In general, the higher X123 energies are better for inferring the hotter plasma conditions.

If the plasma were isothermal, then one would expect an isothermal fit to intersect (or overlap with) the Em loci at the corresponding temperature value. Additionally, the place where the various Em loci curves for different energy bins intersect is roughly the location that yields the isothermal temperature and emission measure that best describe the plasma. In the case of large flares during peak emission times, we expect the flaring plasma to dominate the solar spectral emission. Thus, a 1T model result would peak near this value, and this is what was observed for the 1T models. The 1T-Free model for the QS and three pre-flare times are displayed in Figure 13 to exemplify this (the diamond symbols). The values for all the pre-flare times are near 3 MK and coincide with the GOES/XRS isothermal estimates for coronal (squares) and photospheric (pentagons) abundances. Moreover, the X123 1T-Free values lie in between the GOES/XRS coronal and photospheric estimates, as they should, because the fit results for X123 reside in between coronal and photospheric FIP-bias values. The 3 MK isothermal plasma temperature is consistent with the inferred AR DEM peak from the combined *Hinode*/EIS and SDO/AIA study of Del Zanna (2013). The 2T-AllFree Em loci results are displayed in Figure 14. The best-fit emission measure and temperatures are the delta functions (in temperature) with stars. The Woods *et al.* (2017) M5.0 flare fit results are overlaid as the dashed histograms in the bottom right panel. The thick black Em loci show the M5.0 flare and the thick cyan Em loci the pre-flare; both are the minimum of all the individual energy bins corresponding to the spectral model used in the Woods *et al.* (2017) analysis. The GOES/XRS average flare-peak time 1T emission measure and temperature results for photospheric and coronal elemental abundance are overplotted for comparison.

The flare-peak GOES estimates all have lower temperature, higher emission measure, and lie closer to the region where the MinXSS/X123 Em loci come close to intersecting for the three flare times. The X123 loci straddle the Em loci both above and below the GOES values. 1T fits for X123 during the flare-peak times are similar to the GOES estimates, but are not shown because of the poor fit to the X123 flare spectra. The RHESSI fit results are also plotted for the M1.2 and M5.0 flare. The RHESSI estimates are hotter for the M1.2 flare than the MinXSS components and GOES, and the M5.0 flare is consistent with GOES estimates, but both RHESSI estimates are consistent with the MinXSS Em loci.

The consistency between X123 and XP Em loci provides further confidence that both instruments are performing nominally. The minimum of all the Em loci for the MinXSS-1/X123 energy bins provides an upper limit to any multi-temperature and DEM fits. The energy must be spread over a range of temperatures and thus could not intersect the Em loci at any point. Thus, Em loci provide firm upper limits to the temperature distribution for a particular measurement from a particular instrument. Em loci are not a new tool; they have been used most notably in XRT, EIS, AIA, FOXSI, RHESSI, and NuSTAR analyses, to name a few. Em loci can be a valuable aid in MinXSS data analysis.

## Summary

The MinXSS CubeSats can provide the solar community with a new set of measurements that can augment current and future investigations of the solar corona. The SPS ancillary instrument, the X-ray detectors of XP, and X123 have been characterized. The first version of MinXSS, MinXSS-1, has performed nominally over its mission at LEO. This article described the MinXSS instrument suite, the X123 FOV sensitivity, X123 spectral resolution *vs*. photon energy, XP and X123 effective area curves, X123 detector response matrix, XP and X123 temperature response, the X123 linearity of response, GOES flux levels *vs*. MinXSS-1/X123 measured integrated counts, and MinXSS-2/X123 estimated counts, inferred temperature and emission measures from MinXSS spectra, and emission measure loci for the discussed data. These realizations further the notion that CubeSats can conduct significant targeted science. A summary of the main attributes of MinXSS is listed below.

i) MinXSS-1/X123 has an effective solar flux energy range of 0.8 – 12 keV (${\approx}\,0.10\,\mbox{--} \,1.55~\mbox{nm}$) with a resolving power ${\approx}\,40$ at 5.9 keV, and with dead-time corrections applied, it provides accurate spectra up to low GOES X levels (but they need to be corrected for pulse-pileup).
ii) The X123 on the MinXSS CubeSats have a relatively higher spectral resolution over a fairly broad bandpass, which allows inference of elemental abundance values for the elements Fe, Ca, Si, Mg, S, Ar and Ni, when there are sufficient counts at X123 energies for their respective line-groups. The observed elemental abundance variation in this work
  clearly demonstrates a decrease in low-FIP elements for flare-peak times *vs*. the pre-flare values for spectral fits with a single FIP-bias multiplicative factor
  display variance in the fractionation pattern among the low-FIP elements when all elements are allowed to vary for GOES levels larger than B1.
iii) MinXSS-1/X123 flare measurements in this article indicate that the hotter components in the flaring plasma for the C2.7, M1.2, and M5.0 flares have peak temperatures near 15, 13, and 20 MK, respectively.
iv) MinXSS-1/X123 and XP plasma temperatures inferences are self-consistent, and single X123 temperature fits (1T-Free) are comparable with GOES/XRS isothermal estimates between GOES ${\approx}\,\mbox{B1}\,\mbox{--}\,\mbox{M5}$ levels (the GOES/XRS response becomes nonlinear for lower flux levels). However, these single-temperature fits cannot account for high-energy X123 spectral counts, nor are they suitable fits for an entire flare spectrum alone.
v) MinXSS/X123 can infer non-large-flaring Sun properties between 1.5 – 4 MK with high confidence, but it has limited capabilities for temperatures below 1.5 MK (because of the limited sensitivity at lower energies, ${<}\,1~\mbox{keV}$).
vi) MinXSS/X123 can only set upper limits on the emission measure and cannot definitively constrain the temperature values for dimmer plasma hotter than ${\approx}\,5~\mbox{MK}$ during non-large-flaring times. This is due to the flattening nature of the temperature response above ${\approx}\,4.5~\mbox{MK}$ for energy bins lower than 3 keV and limited significant counts from energy bins greater than 3 keV. The latter is a consequence of the relatively small X123 aperture area ($2.5\times10^{-4}~\mbox{cm}^{2}$).

The anticipated mission length for MinXSS-1 was one year. The second version, MinXSS-2, has improved hardware, software, and detectors (see Section 6.1). The anticipated duration of the Sun-synchronous orbit for MinXSS-2 is five years, providing a possible total of six years of nearly continuous measurements. The MinXSS-1 Level-1 products are available on the MinXSS website: http://lasp.colorado.edu/home/minxss/.

### Improvements of MinXSS-2

The MinXSS-2/X123 has a thinner Be window along with a lower noise preamplifier that will provide an improved low-energy response, possibly extending the low-energy limit to 0.6 keV. The newer preamplifier for the MinXSS-2/X123 Fast SDD detector allows for a better spectral resolution for the same nominal peaking time as the MinXSS-1/X123. We will be operating a shorter peaking time for the MinXSS-2/X123 that will allow for accurate spectra for a higher input flux. Preliminary estimates suggest that GOES levels around X2 are the maximum that the MinXSS-2/X123 can handle before the spectra will need further processing to retain fidelity.

## Appendix: SPS Architecture and Position Calculation

The SPS quad Si-photodiode detector assembly is used to calculate the solar position in the MinXSS FOV. SPS sits behind an ND7 filter (attenuation of a factor of $10^{7}$) to limit the visible-light flux and eliminate all other electromagnetic contributions to the signal. SPS relies on the visible-light emission from the Sun to estimate the solar location within the FOV position by calculating $\alpha$ and $\beta$. Figure 16 shows the basic SPS layout of the quad diode assembly with a square $2 \times 2~\mbox{mm}$ aperture. SPS has no focusing optics and thus its operating principle relies upon measuring the relative light detected on each of the four diodes Q1, Q2, Q3, and Q4 to calculate the solar disk location. Equations 9 and 10 display the relation between the quad diode signal to the $\alpha$ and $\beta$ coordinates, which must be scaled by the known maximum angular deviation $\alpha_{\mathrm{max}}$ and $\beta_{\mathrm{max}}$. The maximum angular deviation is set by the geometrical layout of the aperture with respect to the quad diodes. For an aperture of width, $w$, height, $h$, and separation between the photodiode and aperture along the optical path, $d$, the maximum angles can be calculated from Equations 11 and 12,

9 $$\begin{aligned} \alpha =& \alpha_{\mathrm{max}} \frac{(Q1 + Q4) - (Q2 + Q3)}{(Q1 + Q2 + Q3 + Q4)} , \end{aligned}$$

10 $$\begin{aligned} \beta =& \beta_{\mathrm{max}} \frac{(Q1 + Q2) - (Q3 + Q4)}{(Q1 + Q2 + Q3 + Q4)} , \end{aligned}$$

11 $$\begin{aligned} \alpha_{\mathrm{max}} =& \alpha \tan \biggl(\frac{w}{2d} \biggr) , \end{aligned}$$

12 $$\begin{aligned} \beta_{\mathrm{max}} =& \beta \tan \biggl(\frac{h}{2d} \biggr) . \end{aligned}$$
